# Low-intensity mindfulness and cognitive–behavioral therapy for social anxiety: a pilot randomized controlled trial

**DOI:** 10.1186/s12888-024-05651-0

**Published:** 2024-03-07

**Authors:** Shota Noda, Kentaro Shirotsuki, Mutsuhiro Nakao

**Affiliations:** 1 Department of Psychology, Translational Clinical Psychology, Philipps University Marburg, Schulstraße 12, Marburg, 35032 Germany; 2 Research Institute of Cognitive Behavior Therapy, Musashino University, 3-3-3 Ariake, Koutou-Ku, Tokyo, 135-8181 Japan; 3 Faculty of Human Sciences, Musashino University, 3-3-3 Ariake, Koutou-Ku, Tokyo, 135-8181 Japan; 4 Department of Psychosomatic Medicine, School of Medicine, International University of Health and Welfare, 4-3, Kozunomori, Narita-Shi, Chiba, 286-8686 Japan

**Keywords:** Mindfulness, Cognitive–behavioral therapy, Social anxiety, Low-intensity program, Randomized controlled trial

## Abstract

**Background:**

Cognitive–behavioral therapy (CBT) effectively improves the clinical symptoms of social anxiety disorder. However, there are non-responders who cannot decrease their cost/probability bias significantly; hence, their social anxiety symptoms remain unaddressed. Mindfulness training and cognitive–behavioral approaches promote a reduction in cost/probability bias and social anxiety symptoms. This study examines the effectiveness of a four-session program of mindfulness and CBT (M-CBT) in a non-clinical sample of individuals with high social anxiety.

**Methods:**

Participants were 50 Japanese undergraduate students (37 women and 13 men) randomly allocated to an intervention group (*n* = 27) and a control group (*n* = 23). The intervention group underwent a four-session M-CBT program, while the control group did not receive any treatment.

**Results:**

A group × time analysis of covariances showed significant interactions in the negative cognition generated when paying attention to others in probability bias, fear of negative evaluation by others, dispositional mindfulness, depressive symptoms, and subjective happiness. M-CBT also produced significant pre-post improvements in the above outcomes with moderate to high effect sizes (*d*s = .51–1.55). Conversely, there were no interactions in social anxiety symptoms and self-focused attention.

**Conclusions:**

These results indicate that M-CBT was effective for the negative cognition generated when paying attention to others in probability bias, fear of negative evaluation by others, dispositional mindfulness, depressive symptoms, and subjective happiness. The combination of mindfulness training with cognitive restructuring is proposed as potentially helpful for individuals with probability bias, leading to negative cognition from paying attention to others.

**Trial registration:**

University Hospital Medical Information Network (UMIN CTR) UMIN000036763. Registered May 16, 2019.

**Supplementary Information:**

The online version contains supplementary material available at 10.1186/s12888-024-05651-0.

## Background

Social anxiety disorder (SAD) is characterized by marked or intense fear or anxiety of social situations in which an individual may feel scrutinized by others [1]. Among various SAD interventions, cognitive–behavioral therapy (CBT) is the most effective in addressing its symptoms [2]. The CBT program for SAD comprises techniques such as psychoeducation, self-monitoring, cognitive restructuring, and exposure.

Despite such therapeutic techniques, some patients do not experience clinically significant improvement, even at the end of their treatment [3]. According to Loerinc et al. [4], the response rate for the treatment of SAD through CBT is 45.3%. Springer et al. [5] conducted a meta-analysis to examine the SAD remission rate of patients (aged 18 years or older) with anxiety disorders treated with CBT and found a 40.1 to 40.4% remission rate. The remission rate in patients (aged 7–17 years) with SAD who underwent CBT was 40.6% [6]. This suggests a remission rate of SAD through CBT of approximately 40%. Therefore, developing an intervention that is effective for patients whose symptoms do not improve with traditional CBT is necessary.

Moscovitch et al. [7] compared changes in clinical outcomes at three time points (pre-, mid-, and post-treatment) between patients with SAD who responded to CBT and those who did not and found differences in cost and probability bias changes. Specifically, responders showed a significant decrease in cost and probability biases, while non-responders did not. Cost bias refers to the exaggerated cost (negative valence) associated with negative social events [8]. It is the tendency to view one’s own performance as catastrophic and believe that the worst is to come. Probability bias, however, is an exaggerated estimate of the occurrence of negative social events [8] and the tendency to predict a high likelihood of negative social consequences or events. According to the cognitive–behavioral models of SAD [9, 10], cost/probability bias is a factor in maintaining SAD and exacerbates avoidance behavior and social anxiety. CBT has been known to improve cost/probability bias and social anxiety symptoms; in particular, a reduction in cost bias is a strong predictor of improvement in social anxiety symptoms [8]. Hofmann [11] found higher effect sizes of cost bias and social anxiety symptoms and maintained at follow-up when CBT, including cognitive interventions for cost/probability bias, was used. Conversely, the effect sizes were lower when exposure therapy was employed without explicit cognitive interventions. Hofmann also suggested that early changes in estimated cost bias were associated with delayed changes in social anxiety symptoms among participants undergoing CBT. Moreover, Shirotsuki et al. [12] found that a CBT program targeting cost/probability bias was effective for patients with SAD, emphasizing the importance of reducing cost/probability bias in the treatment for SAD. These studies further exemplify the need to introduce intervention techniques that address cost/probability bias in patients with SAD. Such techniques may also prove beneficial for patients who do not respond to conventional CBT.

Mindfulness training (MT) techniques aim to improve dispositional mindfulness; they are expected to promote the reduction of cost/probability bias [13, 14]. Therapeutic programs constructed with MT as the primary intervention technique or with mindfulness as the core theory are referred to as mindfulness-based interventions (MBIs). MBIs for SAD include mindfulness-based stress reduction (MBSR), mindfulness-based cognitive therapy (MBCT), and mindfulness and acceptance-based group therapy (MAGT). The MBSR program developed by Kabat-Zinn [15] comprises MT strategies such as meditation, body scans, and mindfulness yoga. Goldin et al. [16] found high therapeutic efficacy in patients with SAD who participated in a 12-session MBSR program, with each session lasting 2.5 h. MBCT, based on MBSR [17], was originally developed to prevent the recurrence of depression. Its effectiveness in participants with SAD was demonstrated through eight weekly 2-h sessions [18]. MAGT, developed by Fleming and Kocovski [19], comprises 12 weekly 2-h sessions. The program, based on CBT and acceptance and commitment therapy theory, comprises mindfulness and acceptance and commitment therapy exercises (including exposure). Moreover, it has previously demonstrated high therapeutic efficacy for SAD [20]. A meta-analysis showed that MBIs are less effective than CBT [21]; however, some studies have reported similar effects of MBIs and CBT [16, 22]. MBIs also increase distancing from thoughts, cognitive flexibility, and cognitive reappraisal skills [23, 24]. These techniques are effective in the treatment of negative cognitions and social anxiety symptoms in patients with SAD [16, 18, 25, 26].

Dispositional mindfulness affects social anxiety symptoms in a myriad of ways. Noda et al. [13] found that dispositional mindfulness negatively affects social anxiety through self-focused attention, cost/probability bias, and avoidance behaviors. Moreover, cost bias was directly (indirectly) affected by dispositional mindfulness (self-focused attention and probability bias) in the path model proposed by the authors. MT impacts cognitive reappraisal skills via the enhancement of dispositional mindfulness and mental health outcomes through mediating improvements in repetitive negative thinking [27, 28]. Goldin et al. [16] found that MBIs affected post-intervention social anxiety symptoms by mediating improvements in cognitive distortions. Further, reductions in cognitive distortion mediated the impact of MBIs at the same level as CBT. An MBI, which involves both MT and a cognitive–behavioral approach, is effective against cost bias in patients with SAD [29]. Noda et al. [29] implemented the MBI comprising four sessions and a half-day retreat. They reported reductions in cost bias before the fourth session of the MBI, using the cognitive–behavioral approach. These findings suggest that MT may address the issue of cost bias in patients with SAD.

Further, the suggested short-term effects of MT include increasing the efficacy of cognitive restructuring [27]. Noda and Shirotsuki [30] suggested that because MT promotes thought awareness, cognitive restructuring post-MT may facilitate more constructive thoughts. Barlow et al. [31] cited MT as a technique for cultivating essential skills that enhance patient’s ability to reflect on their treatment progress, indicating that cognitive restructuring post-MT may increase thinking flexibility and help identify unhelpful thoughts. Ito [32] similarly suggested that the effects of CBT can be enhanced by incorporating the concept of mindfulness and its techniques. Heimberg [33] indicated three stages in cognitive restructuring: (a) identifying negative thoughts that occur before, during, or after anxiety-provoking situations; (b) evaluating the accuracy of these thoughts objectively; and (c) deriving rational alternative thoughts based on the acquired information. MT can enhance awareness of one’s thoughts, promote distancing from thoughts, improve cognitive flexibility, and develop cognitive reappraisal skills [17, 23, 24, 34]. Moreover, MT is suggested to enhance the therapeutic effects of cognitive restructuring [30–32]. Therefore, the combined use of cognitive restructuring and MT may augment the efficacy of the former and reduce cognitive biases and related symptoms. Noda et al. [13] also suggested that mindfulness has an important role in improving social anxiety. Additionally, they posit that MT may contribute not only to improving cost/probability bias and social anxiety but also self-focused attention and avoidance behavior, which are maintaining factors for cost/probability and social anxiety. However, the combined impact of MT and cognitive restructuring on cost/probability bias and social anxiety symptoms has not been fully clarified. Therefore, Noda et al. [35] developed a four-session mindfulness CBT (M-CBT) program for social anxiety, which combines MT and cognitive restructuring to address this gap. Significantly, it is hypothesized that MT enhances the efficacy of cognitive restructuring and promotes improvements in social anxiety and cost/probability bias in M-CBT [35].

Noda et al. [29] found that M-CBT was effective for social anxiety symptoms and cost/probability bias in patients with SAD based on a single-arm study; however, to our knowledge, no related studies have been conducted using randomized controlled trials (RCTs) or comparing M-CBT with cognitive restructuring. Thus, generating evidence on the use of mindfulness and cognitive restructuring in treating cost/probability bias and social anxiety symptoms is necessary. As a first step to validate the hypothesis of M-CBT, this study examined the effectiveness of M-CBT in individuals with high social anxiety symptoms using the RCT. The trial involved two groups: an intervention group that underwent M-CBT and a control group that did not receive any treatment.

The program was also designed as a brief, low-intensity treatment module specifically for individuals with mild SAD, targeting those with high levels of cost/probability bias and social anxiety symptoms. Noda et al. [29] found that M-CBT had significant effects on cost/probability bias and social anxiety symptoms in patients with SAD with an average treatment duration of 752 days. The effect size of M-CBT was similar to that of MBIs comprising 8 to 12 sessions [18, 20]. Developing a program with fewer sessions than the traditional program could reduce the cost of treatment and the number of individual visits; thus, the financial burden and inconvenience of receiving treatment might be reduced.

## Methods

### Participants

This study involved Japanese university students with high social anxiety symptoms. The mean onset age of SAD in Japan is 18.6 years [36], with university students showing a high degree of social anxiety symptoms [13]; thus, the age of university students may be the most common age at which SAD symptoms first appear. Previous studies have reported similarities and continuity in social anxiety symptoms between SAD and general population samples [37, 38]. Examining university students with high social anxiety symptoms to determine the effectiveness of M-CBT for social anxiety symptoms may be beneficial. The Liebowitz Social Anxiety Scale (LSAS) is a well-validated scale used to measure the dimensional severity of social anxiety symptoms and determine the presence-absence of SAD [39, 40]. Therefore, the study sample comprised undergraduate students who exceeded the cutoff value of 44 points on the Japanese version of the LSAS [41] for clinical SAD groups in Japan.

Participants were students attending a university in Japan. Inclusion criteria were as follows: (a) a score of 44 or higher (the cutoff for the clinical group in SAD) on the Japanese version of the LSAS [41], (b) a score of 69 or lower (no severe depressive symptoms [42]) on the Japanese version of the Self-rating Depression Scale (SDS [43]), (c) not being under any other psychiatric treatment, and (d) absence severe physical illness. An a priori power calculation by G*Power showed that a sample of at least 26 was required to detect moderate to strong effect size between pre-and post-test or follow-up in the intervention group (*d* = 0.50 to 0.80; alpha = 0.05; power = 0.80).

To recruit participants, we distributed a set of questionnaires comprising the LSAS and SDS to the students attending their university lectures; the ethical considerations and research outline were explained to them in writing and verbally. Ninety-nine individuals agreed to participate; of these, 71 met the inclusion criteria and were randomly assigned to the intervention (*n* = 36) and control (*n* = 35) groups using a computerized random number generator. Participants were notified through email. Fifty participants (27 in the intervention and 23 in the control group) responded to the participation request. Of the remaining 21 participants, three (one in the intervention and two in the control group) declined to participate, and 18 (eight in the intervention and 10 in the control group) did not respond to the email. The students who responded were individually briefed about this study and signed written informed consent forms before enrollment. The demographic data from the screening survey were analyzed to compare the intervention and control groups. No significant difference was found between them (Table 1).

**Table 1:** Comparison of demographic data of the intervention and control groups at screening

	Intervention group (*n* = 27)	Control group (*n* = 23)	*t-value* or* X*^*2*^*-value*	*p*-values
Sex	Women (*n* = 21), men (*n* = 6)	Women (*n* = 16), men (*n* = 7)	.44	.51
Age	19.81 (± .83)	19.61 (± .66)	.96	.34
LSAS	73.04 (± 17.94)	74.74 (± 20.44)	.31	.76
SDS	46.30 (± 6.10)	46.04 (± 7.26)	.13	.89

*LSAS* Liebowitz Social Anxiety Scale, *SDS* Self-rating Depression Scale

This study was registered in the UMIN Clinical Trial Registration System (UMIN: UMIN000036763) and approved by the Research Ethics Committee of the Faculty of Human Sciences, Musashino University (no. 30007).

## Materials

### Primary outcomes

#### The Japanese version of the Liebowitz social anxiety scale

The LSAS [41] is a self-report scale that measures social anxiety and avoidance behavior in social situations [44]. The scale comprises 24 items, each rated on a four-point scale from 0 (*none* on the anxiety scale and *never* on the avoidance behavior scale) to 3 (*severe* on the anxiety scale and *usually* on the avoidance behavior scale). The total score ranged from 0 to 144 (social anxiety, 72; avoidance behavior, 72), with higher scores indicating greater social anxiety symptoms. The LSAS has high internal consistency, test–retest reliability, and factorial and convergent validity [41, 45].

#### Speech cost/probability scale

The Speech Cost/Probability Scale (SCPS) is a self-report scale that assesses cost and probability bias in speech situations in which patients with SAD exhibit excessive anxiety [46]. It comprises 11 items pertaining to cost and probability bias. Eight items assess negative cognition from one’s performance, while three items assess negative cognition generated when paying attention to others. Each item is rated on a five-point scale from 1 (*not at all* in the cost bias scale, and *I don’t think so at all* in the probability bias scale) to 3 (*very much* in the cost bias scale, and *I very much think* so in the probability bias scale). Each total score ranges from 11 to 55, with higher scores indicating greater cost and probability bias. The SCPS has demonstrated high internal consistency and factorial and convergent validity [46].

### Secondary outcomes

#### The Japanese version of the short fear of negative evaluation scale

The Fear of Negative Evaluation Scale (FNE) is a self-report scale comprising 30 true/false items to assess fear of negative evaluation by others [47]. Ishikawa et al. [48] developed the Japanese version of the FNE, which demonstrated high internal consistency, test–retest reliability, factorial validity, and convergent validity [48]. Sasagawa et al. [49] developed the short version of the Japanese FNE (SFNE) that has two factors (forward-item and reversed-item factor) and contains 12 items rated on a five-point scale from 1 (*not at all characteristic*) to 5 (*extremely characteristic of me*). The total score ranges from 12 to 60, with higher scores indicating greater fear of negative evaluation [49, 50]. The SFNE also has high internal consistency, test–retest reliability, and factorial and convergent validity [50].

#### The Japanese version of the self-focused attention scale

The Self-Focused Attention Scale (SFA) is a self-report scale that measures self-focused attention [51]. It comprises 11 items: six (five) for the arousal (behavior) factor. Each item is rated on a five-point scale from 0 (*not at all*) to 4 (*very much*). The total score ranges from 0 to 44, with higher scores indicating greater self-focused attention. Noda et al. [52] developed the Japanese version of the SFA and reported high internal consistency and factorial, convergent, and discriminant validity.

### Additional outcomes

#### The Japanese version of the five facet mindfulness questionnaire

The Five Facet Mindfulness Questionnaire (FFMQ) is a self-report scale for dispositional mindfulness [53]. It has five subscales: “observing (eight items),” “acting with awareness (eight),” “nonjudging (seven),” “nonreactivity (eight),” and “describing (eight),” each rated on a five-point scale from 1 (*never or very rarely true*) to 5 (*very often or always true*). The total score ranges from 39 to 195, with higher scores indicating greater dispositional mindfulness. Sugiura et al. [54] developed the Japanese version, with acceptable internal consistency and factorial and convergent validity.

#### The Japanese version of the self-rating depression scale

The SDS is a self-reported scale [55] containing 20 items rated on a four-point scale from 1 (*a little of the time*) to 4 (*most of the time*). The total score ranges from 20 to 80, with higher scores indicating greater depressive symptoms. Fukuda and Kobayashi [43] developed the Japanese version of the SDS, having high internal consistency and test–retest reliability [43, 56]. The SDS score was significantly higher in patients with depression than in those with neurosis and the general adult population, suggesting that the SDS has clinical validity [43].

#### The Japanese version of the subjective happiness scale

The Subjective Happiness Scale (SHS) is a self-reported scale to assess subjective happiness, containing four items rated on a seven-point scale [57]. Item 4 is a reversal item. The total score for all items was calculated. The score ranges from 4 to 28, with higher scores indicating greater subjective happiness. Shimai et al. [58] developed the Japanese version of the SHS, which has high internal consistency, test–retest reliability, and factorial, convergent, and discriminant validity.

### Procedure

**Fig. 1:**
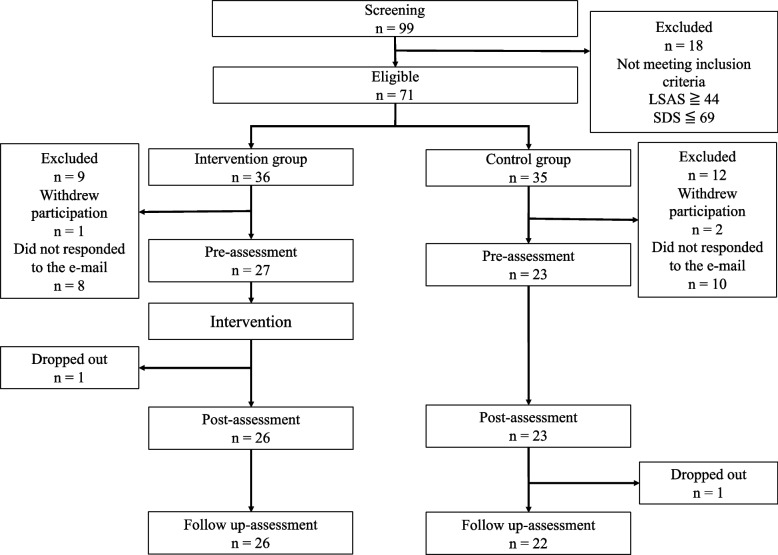
Flowchart showing the process of each stage of a parallel randomized controlled trial between intervention and control groups. Note. LSAS = Liebowitz Social Anxiety Scale; SDS = Self-Rating Depression Scale

Table 2 provides an overview of the program comprising four 90-min sessions delivered once a week in a group format (of three to eight participants each). Detailed information on the M-CBT protocol, the purpose of M-CBT, an overview of the four-session program, homework, and place and therapist is provided in Supplementary Material 1. The intervention group underwent the four-session M-CBT program. Participants were asked to self-report any negative physical or mental changes before each session. Questionnaires were administered to the participants before and immediately after the intervention and one month later. The questionnaire surveys were conducted individually in a room with a therapist. At this time, participants were asked about their physical and mental condition and assessed for any negative physical or mental changes resulting from participation in the program. Participants were also provided with the therapist’s email address and instructed to contact them if they experienced any negative changes in their physical and mental condition. However, none reported any negative physical or mental changes. One participant withdrew after the first session because they had to attend other activities at university. The control group did not participate in the intervention program, nor did it undergo any other interventions; however, the questionnaires were administered to the control group with timing and sequence identical to those administered to the intervention group. As with the intervention group, the questionnaire surveys were conducted individually in a room with a therapist. Figure 1 presents a flowchart of the parallel RCT process.

**Table 2:** Overview of the M-CBT protocol

Session	Title	Intervention techniques	Homework
1	Discover the factors that are increasing social anxiety	Mindfulness yoga Developing a treatment plan Psychoeducation Sitting meditation Sharing	Sitting meditation Diary of daily happiness
2	Identify the factors that are causing social anxiety	Mindfulness yoga Psychoeducation Imagery mediation Sharing	Sitting or imagery meditation Diary of thoughts, emotions, behaviors, and physical reactions in interaction situations with others
3	Observe the factors that are causing social anxiety	Developing an anxiety hierarchy list and personal version of cognitive–behavioral models Imagery mediation Sharing	Sitting or imagery meditation Diary of communication with others
4	Let go of the factors that are causing social anxiety	Imagery meditation Cognitive restructuring Loving-kindness meditation Sharing	

*M-CBT* mindfulness and Cognitive Behavioral therapy

### Statistical analyses

A Kolmogorov–Smirnov normality test was performed to examine the normality of the distribution for each scale. The Kolmogorov–Smirnov normality test results are shown in Supplementary Material 2. The normality of the distribution was assumed for many variables (*p* > 0.05). Therefore, we proceeded to examine the effect of M-CBT using parametric tests.

Interactions between the intervention and control groups were analyzed with a 2 (group: intervention and control groups) × 2 (time: pre-and post-test) and 3 (time: pre-test, post-test, and follow-up test) analysis of covariances (ANCOVA) to test the effectiveness of M-CBT, with pre-test scores of the respective measures used as covariates. In addition, simple effect analyses using Bonferroni’s method were performed for variables for which an interaction was confirmed. Conversely, for variables for which no interaction was found, main effects between and within groups were examined, and multiple comparisons using Bonferroni’s method were conducted when significant main effects were shown. Cohen’s *d* was calculated to examine the effect size within the intervention group. According to Cohen [59], Cohen’s *d *of approximately 0.20 is small; 0.50, medium; and 0.80, large, and is considered significant when the lower and upper confidence intervals do not cross zero. Statistical software SPSS version 28.0 (IBM Corp., Armonk, NY, USA) was used for ANCOVAs and effect sizes.

In Koszycki et al. [60], the sample of patients who attended at least 80% of the intervention program sessions was considered a complete sample. In this study, individuals in the intervention group (intent-to-treat analyses [ITT]) and individuals in the intervention group who participated in more than three-fourths of the sessions (per-protocol analyses [PPA]) were considered for the analyses. Of the 27 participants in pre-assessment, 22 completed four sessions of the program, and four completed three sessions; thus, 26 individuals were included in the PPA. One participant who withdrew was not included in the post-assessment. In the control group, 23 participants were included in the ITT. Since the one participant who dropped out was not included in the follow-up assessment, the PPA involved 23 participants for the group × time (2 × 2) ANCOVA and 22 participants for the group × time (2 × 3) ANCOVA. Missing values in the ITT were complemented by the last observation carried forward.

## Results

### ITT sample

Table 3 shows the means and standard deviations of the outcome indicators of each group and the results of ANCOVA in the ITT. Detailed information on the results and changes for each variable in the ITT are shown in Supplementary Material 3.

**Table 3:** Means and standard deviations of outcomes in each group and results of ANCOVAs

**ITT sample**			**Intervention group**			**Control group**		Group × 2Times	Group × 3Times
			*Mean*	*SD*		*Mean*	*SD*	*F-value*	*F-value*
**Primary outcomes**									
LSAS total score	Pre-test	*n* = 27	67.22	23.01	*n* = 23	70.70	23.54	.29	.66
	Post-test	*n* = 27	61.11	20.04	*n* = 23	66.04	25.92		
	Follow-up	*n* = 27	61.56	18.60	*n* = 23	69.09	27.89		
LSAS anxiety	Pre-test	*n* = 27	39.33	12.95	*n* = 23	39.43	11.63	.18	.43
	Post-test	*n* = 27	36.22	12.06	*n* = 23	37.39	13.72		
	Follow-up	*n* = 27	36.00	10.88	*n* = 23	38.30	14.47		
LSAS avoidance behavior	Pre-test	*n* = 27	27.89	11.02	*n* = 23	31.26	12.79	.46	.80
	Post-test	*n* = 27	24.89	9.06	*n* = 23	28.65	13.27		
	Follow-up	*n* = 27	25.56	9.24	*n* = 23	30.78	14.36		
SCPS cost bias total score	Pre-test	*n* = 27	34.22	9.05	*n* = 23	33.43	7.29	.34	1.96
	Post-test	*n* = 27	31.81	7.83	*n* = 23	32.48	7.99		
	Follow-up	*n* = 27	29.44	7.64	*n* = 23	32.39	8.34		
SCPS cost bias Negative cognition from one’s performance	Pre-test	*n* = 27	22.96	7.81	*n* = 23	22.57	5.47	.02	1.57
	Post-test	*n* = 27	22.30	6.15	*n* = 23	21.87	6.47		
	Follow-up	*n* = 27	20.26	5.97	*n* = 23	22.09	6.91		
SCPS cost bias Negative cognition generated when paying attention to others	Pre-test	*n* = 27	11.26	2.01	*n* = 23	10.87	2.49	3.64	2.69
	Post-test	*n* = 27	9.52	2.53	*n* = 23	10.61	2.15		
	Follow-up	*n* = 27	9.19	2.34	*n* = 23	10.30	2.38		
SCPS probability bias total score	Pre-test	*n* = 27	31.00	8.47	*n* = 23	31.26	8.22	8.90^**^	3.93^*^
	Post-test	*n* = 27	24.52	6.69	*n* = 23	29.52	7.38		
	Follow-up	*n* = 27	25.89	8.45	*n* = 23	25.89	8.45		
SCPS probability bias Negative cognition from one’s performance	Pre-test	*n* = 27	20.59	6.76	*n* = 23	21.04	6.48	3.60	1.84
	Post-test	*n* = 27	17.15	5.14	*n* = 23	19.91	6.15		
	Follow-up	*n* = 27	17.81	6.31	*n* = 23	18.70	5.97		
SCPS probability bias Negative cognition generated when paying attention to others	Pre-test	*n* = 27	10.41	2.19	*n* = 23	10.22	2.70	19.04^**^	7.86^**^
	Post-test	*n* = 27	7.37	1.92	*n* = 23	9.61	2.43		
	Follow-up	*n* = 27	8.07	2.64	*n* = 23	9.26	2.07		
**Secondary outcomes**									
SFA total score	Pre-test	*n* = 27	27.26	8.34	*n* = 23	25.09	7.69	.12	.38
	Post-test	*n* = 27	24.78	7.58	*n* = 23	23.96	7.48		
	Follow-up	*n* = 27	22.04	8.01	*n* = 23	22.52	7.90		
SFA arousal	Pre-test	*n* = 27	13.48	5.60	*n* = 23	11.22	4.75	.13	.22
	Post-test	*n* = 27	12.26	4.06	*n* = 23	10.39	5.98		
	Follow-up	*n* = 27	10.89	5.48	*n* = 23	9.91	5.89		
SFA behavior	Pre-test	*n* = 27	13.78	4.20	*n* = 23	13.87	4.04	1.56	1.26
	Post-test	*n* = 27	12.52	4.23	*n* = 23	13.57	3.23		
	Follow-up	*n* = 27	11.15	4.35	*n* = 23	12.61	3.65		
SFNE total score	Pre-test	*n* = 27	44.22	8.46	*n* = 23	44.96	7.26	6.46^*^	8.91^**^
	Post-test	*n* = 27	38.74	8.47	*n* = 23	44.39	9.14		
	Follow-up	*n* = 27	37.00	7.36	*n* = 23	44.17	6.77		
SFNE forward-item	Pre-test	*n* = 27	28.04	6.60	*n* = 23	29.13	5.50	6.38^*^	8.22^**^
	Post-test	*n* = 27	24.78	6.20	*n* = 23	29.00	5.98		
	Follow-up	*n* = 27	23.00	5.99	*n* = 23	28.39	5.16		
SFNE reversed-item	Pre-test	*n* = 27	16.19	2.50	*n* = 23	15.83	2.90	4.78^*^	5.40^**^
	Post-test	*n* = 27	13.96	2.82	*n* = 23	15.39	3.54		
	Follow-up	*n* = 27	14.00	2.24	*n* = 23	15.78	2.43		
**Additional outcomes**									
FFMQ	Pre-test	*n* = 27	111.22	9.92	*n* = 23	112.30	13.29	33.27^**^	22.29^**^
	Post-test	*n* = 27	124.11	13.30	*n* = 23	108.74	10.09		
	Follow-up	*n* = 27	126.70	16.09	*n* = 23	110.04	9.91		
FFMQ	Pre-test	*n* = 26	112.00	9.24	*n* = 23	112.30	13.29	39.54^**^	24.16^**^
SDS	Pre-test	*n* = 27	45.07	6.25	*n* = 23	43.13	8.70	13.31^**^	10.54^**^
	Post-test	*n* = 27	39.59	7.38	*n* = 23	44.30	9.00		
	Follow-up	*n* = 27	38.67	8.22	*n* = 23	43.26	8.46		
SHS	Pre-test	*n* = 27	17.48	3.90	*n* = 23	17.13	3.99	17.56^**^	13.15^**^
	Post-test	*n* = 27	20.78	3.71	*n* = 23	17.09	3.44		
	Follow-up	*n* = 27	20.00	3.51	*n* = 23	17.13	3.55		

*ANCOVAs* analysis of covariances, *FFMQ* Five Facet Mindfulness Questionnaire, *SCPS* Speech Cost/Probability Bias Scale, *SFNE* Short Fear of Negative Evaluation Scale, *SDS* Self-rating Depression Scale, *SFA* Self-focused Attention Scale, *SHS* Subjective Happiness Scale, *LSAS* Liebowitz Social Anxiety Scale

^**^p < .01

^*^p < .05

#### Primary outcomes

The results of the group × time (2 × 2) ANCOVA showed significant interactions in the SCPS Probability bias total score and SCPS Probability bias in the negative cognition generated when paying attention to others (*p* < 0.01). Conversely, no interactions were observed in the LSAS total score (*p* = 0.59), LSAS anxiety (*p* = 0.67), LSAS avoidance behavior (*p* = 0.50), SCPS Cost bias total score (*p* = 0.56), SCPS Cost bias in the negative cognition from one’s performance (*p* = 0.88), SCPS Cost bias in the negative cognition generated when paying attention to others (*p* = 0.06), and SCPS Probability bias in the negative cognition from one’s performance (*p* = 0.06).

The results of the group × time (2 × 3) ANCOVA showed significant interactions in the SCPS Probability bias total score and SCPS Probability bias in the negative cognition generated when paying attention to others (*p* < 0.01). However, no interactions were noted in the LSAS total score (*p* = 0.52), LSAS anxiety (*p* = 0.66), LSAS avoidance behavior (*p* = 0.46), SCPS Cost bias total sore (*p* = 0.15), SCPS Cost bias in the negative cognition from one’s performance (*p* = 0.21), SCPS Cost bias in the negative cognition generated when paying attention to others (*p* = 0.07), and SCPS Probability bias in the negative cognition from one’s performance (*p* = 0.16).

#### Secondary outcomes

The results of the group × time (2 × 2) ANCOVA showed significant interactions in the SFNE total score, SFNE forward-item, and SFNE reversed-item (*p* < 0.01). Conversely, no interactions were observed in the SFA total score (*p* = 0.73), SFA arousal (*p* = 0.72), and SFA behavior (*p* = 0.22).

The results of the group × time (2 × 3) ANCOVA showed significant interactions in the SFNE total score, SFNE forward-item, and SFNE reversed-item (*p* < 0.01). However, no interactions were found in the SFA total score (*p* = 0.69), SFA arousal (*p* = 0.80), and SFA behavior (*p* = 0.29).

#### Additional outcomes

The results of the group × time (2 × 2) ANCOVA showed significant interactions in the FFMQ, SDS, and SHS (*p* < 0.01).

The results of the group × time (2 × 3) ANCOVA showed significant interactions in the FFMQ, SDS, and SHS (*p* < 0.01).

### PPA sample

Table 4 shows the means and standard deviations of the outcome indicators of each group and the results of ANCOVA in the PPA. Detailed information on the results and changes in each variable in the PPA are shown in Supplementary Material 4.

**Table 4:** Means and standard deviations of outcomes in each group and results of aNCOVAs

**PPA sample**			**Intervention group**			**Control group**		Group × 2Times	Group × 3Times
			*Mean*	*SD*		*Mean*	*SD*	*F-value*	*F-value*
**Primary outcomes**									
LSAS total score	Pre-test	*n* = 26	67.23	23.46	*n* = 23	70.70	23.54	.33	.64
	Post-test	*n* = 26	60.88	20.40	*n* = 23	66.04	25.92		
	Follow-up	*n* = 26	61.35	18.94	*n* = 22	69.59	28.44		
LSAS anxiety	Pre-test	*n* = 26	39.35	13.20	*n* = 23	39.43	11.63	.21	.39
	Post-test	*n* = 26	36.12	12.28	*n* = 23	37.39	13.72		
	Follow-up	*n* = 26	35.88	11.08	*n* = 22	38.50	14.78		
LSAS avoidance behavior	Pre-test	*n* = 26	27.88	11.24	*n* = 23	31.26	12.79	.51	.82
	Post-test	*n* = 26	24.77	9.22	*n* = 23	28.65	13.27		
	Follow-up	*n* = 26	25.46	9.41	*n* = 22	31.09	14.62		
SCPS cost bias total score	Pre-test	*n* = 26	33.88	9.06	*n* = 23	33.43	7.29	.50	1.88
	Post-test	*n* = 26	31.38	7.65	*n* = 23	32.48	7.99		
	Follow-up	*n* = 26	28.92	7.29	*n* = 22	32.14	8.44		
SCPS cost bias Negative cognition from one’s performance	Pre-test	*n* = 26	22.65	7.80	*n* = 23	22.57	5.47	.00	1.52
	Post-test	*n* = 26	21.96	6.02	*n* = 23	21.87	6.47		
	Follow-up	*n* = 26	19.85	5.68	*n* = 22	21.91	7.02		
SCPS cost bias Negative cognition generated when paying attention to others	Pre-test	*n* = 26	11.23	2.05	*n* = 23	10.87	2.49	4.09^*^	2.57
	Post-test	*n* = 26	9.42	2.53	*n* = 23	10.61	2.15		
	Follow-up	*n* = 26	9.08	2.31	*n* = 22	10.23	2.41		
SCPS probability bias total score	Pre-test	*n* = 26	30.65	8.44	*n* = 23	31.26	8.22	11.11^**^	4.27^*^
	Post-test	*n* = 26	23.92	6.05	*n* = 23	29.52	7.38		
	Follow-up	*n* = 26	25.35	8.12	*n* = 22	27.73	7.65		
SCPS probability bias Negative cognition from one’s performance	Pre-test	*n* = 26	20.27	6.68	*n* = 23	21.04	6.48	4.71^*^	2.09
	Post-test	*n* = 26	16.69	4.65	*n* = 23	19.91	6.15		
	Follow-up	*n* = 26	17.38	6.20	*n* = 22	18.55	6.06		
SCPS probability bias Negative cognition generated when paying attention to others	Pre-test	*n* = 26	10.38	2.23	*n* = 23	10.22	2.70	21.95^**^	7.94^**^
	Post-test	*n* = 26	7.23	1.82	*n* = 23	9.61	2.43		
	Follow-up	*n* = 26	7.96	2.63	*n* = 22	9.18	2.08		
**Secondary outcomes**									
SFA total score	Pre-test	*n* = 26	27.12	8.47	*n* = 23	25.09	7.69	.19	.35
	Post-test	*n* = 26	24.54	7.62	*n* = 23	23.96	7.48		
	Follow-up	*n* = 26	21.69	7.96	*n* = 22	22.23	7.95		
SFA arousal	Pre-test	*n* = 26	13.50	5.71	*n* = 23	11.22	4.75	.10	.23
	Post-test	*n* = 26	12.23	4.14	*n* = 23	10.39	5.98		
	Follow-up	*n* = 26	10.81	5.57	*n* = 22	9.68	5.92		
SFA behavior	Pre-test	*n* = 26	13.62	4.20	*n* = 23	13.87	4.04	1.90	1.30
	Post-test	*n* = 26	12.31	4.16	*n* = 23	13.57	3.23		
	Follow-up	*n* = 26	10.88	4.21	*n* = 22	12.55	3.73		
SFNE total score	Pre-test	*n* = 26	43.85	8.39	*n* = 23	44.96	7.26	7.39^**^	9.57^**^
	Post-test	*n* = 26	38.15	8.05	*n* = 23	44.39	9.14		
	Follow-up	*n* = 26	36.35	6.66	*n* = 22	44.23	6.92		
SFNE forward-item	Pre-test	*n* = 26	27.69	6.47	*n* = 23	29.13	5.50	7.58^**^	8.77^**^
	Post-test	*n* = 26	24.31	5.82	*n* = 23	29.00	5.98		
	Follow-up	*n* = 26	22.46	5.40	*n* = 22	28.41	5.28		
SFNE reversed-item	Pre-test	*n* = 26	16.15	2.54	*n* = 23	15.83	2.90	5.24^*^	5.81^**^
	Post-test	*n* = 26	13.85	2.81	*n* = 23	15.39	3.54		
	Follow-up	*n* = 26	13.88	2.20	*n* = 22	15.82	2.48		
**Additional outcomes**									
FFMQ	Pre-test	*n* = 26	112.00	9.24	*n* = 23	112.30	13.29	39.54^**^	24.16^**^
	Post-test	*n* = 26	125.38	11.76	*n* = 23	108.74	10.09		
	Follow-up	*n* = 26	128.08	14.71	*n* = 22	110.18	10.12		
FFMQ	Pre-test	*n* = 26	112.00	9.24	*n* = 23	112.30	13.29	39.54^**^	24.16^**^
SDS	Pre-test	*n* = 26	44.65	5.97	*n* = 23	43.13	8.70	14.61^**^	11.92^**^
	Post-test	*n* = 26	38.96	6.74	*n* = 23	44.30	9.00		
	Follow-up	*n* = 26	38.00	7.60	*n* = 22	43.73	8.35		
SHS	Pre-test	*n* = 26	17.61	3.91	*n* = 23	17.13	3.99	20.11^**^	14.34^**^
	Post-test	*n* = 26	21.04	3.53	*n* = 23	17.09	3.44		
	Follow-up	*n* = 26	20.23	3.36	*n* = 22	17.05	3.61		

*ANCOVAs* analysis of covariances, *FFMQ* Five Facet Mindfulness Questionnaire, *SCPS* Speech Cost/Probability Bias Scale, *SFNE* Short Fear of Negative Evaluation Scale, *SDS* Self-rating Depression Scale, *SFA* Self-focused Attention Scale, *SHS* Subjective Happiness Scale, *LSAS* Liebowitz Social Anxiety Scale

^**^p < .01

^*^p < .05

#### Primary outcomes

The results of the group × time (2 × 2) ANCOVA showed significant interactions in the SCPS Cost bias in the negative cognition generated when paying attention to others, SCPS Probability bias total score, SCPS Probability bias in the negative cognition from one’s performance, and SCPS Probability bias in the negative cognition generated when paying attention to others (*p* < 0.01). Conversely, no interactions were observed in the LSAS total score (*p* = 0.57), LSAS anxiety (*p* = 0.65), LSAS avoidance behavior (*p* = 0.48), SCPS Cost bias total score (*p* = 0.48), and SCPS Cost bias in the negative cognition from one’s performance (*p* = 0.98).

The results of the group × time (2 × 3) ANCOVA showed significant interactions in the SCPS Probability bias total score and SCPS Probability bias in the negative cognition generated when paying attention to others (*p* < 0.01). However, no interactions were evident in the LSAS total score (*p* = 0.53), LSAS anxiety (*p* = 0.68), LSAS avoidance behavior (*p* = 0.44), the SCPS Cost bias total sore (*p* = 0.16), SCPS Cost bias in the negative cognition from one’s performance (*p* = 0.23), SCPS Cost bias in the negative cognition generated when paying attention to others (*p* = 0.08), and SCPS Probability bias in the negative cognition from one’s performance (*p* = 0.13).

#### Secondary outcomes

The results of the group × time (2 × 2) ANCOVA showed significant interactions in the SFNE total score, SFNE forward-item, and SFNE reversed-item (*p* < 0.01). Conversely, no interactions were observed in the SFA total score (*p* = 0.19), SFA arousal (*p* = 0.75), and SFA behavior (*p* = 0.18).

The results of the group × time (2 × 3) ANCOVA showed significant interactions in the SFNE total score, SFNE forward-item, and SFNE forward-item (*p* < 0.01). However, no interactions in the SFA total score (*p* = 0.71), SFA arousal (*p* = 0.80), and SFA behavior (*p* = 0.28).

#### Additional outcomes

The results of the group × time (2 × 2) ANCOVA showed significant interactions in the FFMQ, SDS, and SHS (*p* < 0.01).

The results of the group × time (2 × 3) ANCOVA showed significant interactions in the FFMQ, SDS, and SHS (*p* < 0.01).

### Within-group effect sizes

Table 5 shows the effect sizes between pre-and post-test or follow-up in the ITT and PPA.

**Table 5:** Within-group effect sizes for each outcome

	ITT sample (*n* = 27)				PPA sample (*n* = 26)			
	Pre-post effect sizes (Cohen’s *d*)	95% CI	Pre-follow-up effect sizes (Cohen’s *d*)	95% CI	Pre-post effect sizes (Cohen’s *d*) *n* = 26	95% CI	Pre-follow-up effect sizes (Cohen’s *d*) *n* = 26	95% CI
LSAS total score	.28	-.13–.69	.27	-.11–.64	.29	-.11–.68	.28	-.12–.67
Anxiety	.25	-.12–.62	.27	-.05–.60	.25	-.14–.64	.28	-.11–.67
Avoidance behavior	.30	-.15–.74	.23	-.22–.67	.30	-.09–.69	.23	-.16–.62
SCPS cost bias total score	.28	-.14–.71	.56^*^	.18–.95	.30	-.10–.69	.60^*^	.18–1.02
Negative cognition from one’s performance	.10	-.32–.50	.37^*^	.06–.69	.10	-.29–.48	.41^*^	.01–.81
Negative cognition generated when paying attention to others	.76^*^	.17–1.35	.95^*^	.36–1.54	.79^*^	.34–1.22	.99^*^	.51–1.45
SCPS probability bias total score	.84^*^	.37–1.31	.60^*^	.13–1.08	.92^*^	.45–1.37	.64^*^	.21–1.06
Negative cognition from one’s performance	.56^*^	.15–.98	.42	-.02–.87	.62^*^	.20–1.04	.45^*^	.05–.85
Negative cognition generated when paying attention to others	1.47^*^	.85–2.09	.96^*^	.43–1.49	1.55^*^	.97–2.12	.99^*^	.52–1.46
SFA total score	.31	-.06–.68	.64^*^	.16–1.12	.32	-.08–.71	.66^*^	.23–1.08
Arousal	.24	-.12–.61	.47^*^	.04–.89	.26	-.14–.64	.48^*^	.07–.88
Behavior	.30	-.02–.62	.62^*^	.20–1.04	.31	-.08–.70	.65^*^	.22–1.07
SFNE total score	.65^*^	.20–1.10	.91^*^	.44–1.37	.69^*^	.26–1.12	.99^*^	.51–1.46
Forward-item	.51^*^	.08–.93	.80^*^	.37–1.22	.52^*^	.10–.92	.91^*^	.44–1.36
Reversed-item	.80^*^	.36–1.23	.78^*^	.34–1.21	.83^*^	.38–1.27	.80^*^	.35–1.24
FFMQ	-1.08^*^	-1.62–-.55	-1.14^*^	-1.72–-.56	-1.27^*^	-1.78–-.74	-1.31^*^	-1.83–-.78
SDS	.80^*^	.33–1.27	.86^*^	.40–1.32	.89^*^	.43–1.34	.97^*^	.50–1.44
SHS	-.87^*^	-1.34–-.40	-.68^*^	-1.09–-.26	-.92^*^	-1.37–-.45	-.72^*^	-1.14–-.28

*95% CI* 95% confidence interval, *FFMQ* Five Facet Mindfulness Questionnaire, *SCPS* Speech Cost/Probability Bias Scale, *SFNE* Short Fear of Negative Evaluation Scale, *SDS* Self-rating Depression Scale, *SFA* Self-focused Attention Scale, *SHS* Subjective Happiness Scale, *LSAS* Liebowitz Social Anxiety Scale

^*^p < .05

## Discussion

This study examined the effectiveness of M-CBT in treating individuals with high social anxiety. The primary outcomes of this study were social anxiety symptoms, cost bias, and probability bias. The results of the ANCOVAs in the ITT showed significant interactions in overall probability bias and the negative cognition generated when paying attention to others with respect to probability bias. The results of the ANCOVAs in the PPA showed significant interactions in the negative cognition generated when paying attention to others as part of cost bias, overall probability bias, the negative cognition from one’s performance, and the negative cognition generated when paying attention to others with respect to probability bias. The intervention group showed significant improvement in these variables compared with the control group. Further, moderate to high values were also obtained for the effect size for these variables.

The four-session M-CBT program comprised psychoeducation, MT, cognitive restructuring, and sharing. In the psychological education session, the role of cost/probability bias in social anxiety and the effects of mindfulness were explained, and an individual model using these negative thoughts was derived. MT was used to increase awareness of one’s negative thoughts in anxiety-provoking situations, distance oneself from those thoughts, and enhance the skill of letting go of such thoughts. In cognitive restructuring, participants examined the evidence against their negative thoughts and discovered objective thinking in social situations. In the sharing session, participants shared their experiences and listened to the experiences of others. Through these processes, participants became aware of their negative thoughts caused by the attention of others. Given this, participants could view the situation objectively, which may improve the negative cognition generated when paying attention to others.

However, no significant differences were observed between the intervention and control groups in overall cost bias, negative cognition over one’s performance in cost bias, and social anxiety symptoms. The intervention group did not show significant improvement in overall cost bias and social anxiety symptoms compared to the control group. Although this program hypothesized that the combination of MT and cognitive restructuring would be effective for social anxiety and cost bias, the current results did not show efficacy regarding these variables in M-CBT. Noda et al. [29] examined the effect of M-CBT for patients with SAD and found that M-CBT is effective for treating social anxiety symptoms and the negative cognition from one’s performance, yielding large effect size gains (social anxiety symptoms as measured by LSAS: *d* = 1.04 to 1.06, SCPS Probability bias in the negative cognition from one’s performance: *d* = 0.82 to 1.04, SCPS Probability bias in the negative cognition from one’s performance:* d* = 1.00 to 1.14). Since expectancy for change during treatment is a predictor of treatment effects for social anxiety [3, 61], the treatment motivation might be a factor that differs between our results and those reported by Noda et al. [29]. In the MBIs, treatment motivation is also an important factor in the effectiveness of treatment [15, 62]. Participants were university students with LSAS total scores of 44 or higher at screening. The mean LSAS total score in the intervention group was 73.04 (SD ± 17.94), and the mean LSAS total score in the control group was 74.74 (SD ± 20.44), which is higher than that for patients with mild SAD (51.2 ± 10.5) reported by Asakura et al. [41]. However, the university students in this study were not diagnosed with SAD, attended university lectures, and might not have experienced any difficulties in social functioning. Conversely, patients in the study conducted by Noda et al. [29] were patients with SAD with an average duration of treatment of 752 days. Therefore, it is possible that participants in the current study had a lower motivation for work aimed at improving social anxiety symptoms than those in the study reported by Noda et al. [29]. However, the present study did not measure participant motivation for treatment. Thus, future studies should examine the effect of motivation for treatment on social anxiety-related outcomes.

Another reason might be the small number of sessions in this program. Goldin et al. [16] reported a reduction of 14 or more points as a reliable change in LSAS total scores. A reduction of more than 14 points was evident in the total LSAS scores of 10 participants after the intervention (reliable change response rate: 38.46%). Of these 10 individuals who showed a reliable change, four (15.38%) had LSAS total scores below 44 (cutoff for the clinical group). Conversely, the LSAS total scores of four other participants increased by more than 14 points after the intervention. Similar variability in treatment response was found in the scores of self-focused attention and cost and probability bias. The four-session M-CBT program corresponds to four stages: (a) becoming aware of one’s reaction patterns, such as thoughts, emotions, and body sensations; (b) accepting these reactions; (c) letting go of one’s negative thoughts; and (d) viewing things from an objective perspective. Given that the program ended when participants’ awareness of their thoughts, feelings, and body sensations had increased, it is possible that elevated clinical symptoms persisted among some participants. In future studies, a longer intervention period for the M-CBT program should be used to examine its effectiveness further.

The negative cognition from one’s performance with regard to cost bias did not show sufficient improvement. The program used a thought record for cognitive restructuring but did not objectively observe actual self-performance and reconstruction of negative self-images. This suggests that individuals who underwent the program might not have sufficiently improved the negative cognition from their performances with regard to cost bias. Video feedback, a therapeutic method that involves making a video of one’s performance and reducing distorted self-perception through the feedback from watching it, may be effective in reducing the negative cognition for this factor [10]. Noda et al. [46] reported that the negative cognition over one’s performance in terms of cost bias was a stronger predictor of social anxiety symptoms than the negative cognition generated when paying attention to others. The addition of video feedback to the program may thus improve social anxiety symptoms by reducing negative cognition about oneself in cost bias.

The secondary outcomes of this study were the fear of negative evaluation by others and self-focused attention. ANCOVA results showed a significant interaction in the fear of negative evaluation by others; thus, the intervention group showed significant improvement in the fear of negative evaluation. The fear of negative evaluation is a symptom of SAD [1] and a core maintaining factor in SAD [9, 63]. Improvement in this symptom has been found to be a significant predictor of the response to CBT for SAD. Moderate to strong effect sizes were also obtained between pre-and post-test or follow-up. Therefore, M-CBT improved fear of negative evaluation by others, with its treatment effect maintained up to one month later. However, no interactions were observed in self-focused attention; thus, the intervention group did not show significant improvement in self-focused attention. Self-focused attention is a maintaining factor of SAD [10, 64]; self-focused attention has been suggested to potentially improve through an MBI comprising MT and attention training in threatening social situations [65]. Contrastingly, a brief mindfulness practice was found to increase self-focused attention [66]. The awareness (acceptance) component of mindfulness is negatively (positively) related to self-focused attention [67]. MT cultivates awareness and subsequently increases acceptance; however, acceptance may not have increased in this study owing to the small number of sessions in our program. Consequently, self-focused attention may not have improved.

The additional outcomes of this study were dispositional mindfulness, depressive symptoms, and subjective happiness. We found dispositional mindfulness to be significantly strengthened in the intervention group compared with the control group. A fairly strong effect size was also calculated with a Cohen’s d greater than 1.2 between pre-and post-test or follow-up in the PPA, indicating that the treatment effect was maintained up to one month later. This result suggests that the program can enhance dispositional mindfulness. We also found significantly improved depressive symptoms and subjective happiness in the intervention group compared with the control group. Moderate to high values were also obtained for the effect size in these variables. Dispositional mindfulness is negatively associated with depressive symptoms and positively associated with subjective happiness [68]. MT and loving-kindness meditation are effective in improving positive emotions and depressive symptoms [69–71]. Thus, this program may be effective in improving depressive symptoms and subjective happiness.

Further limitations of this study and future tasks should be considered. Although previous studies have hypothesized that the combined use of MT with cognitive restructuring would enhance the latter [29, 35], the present study did not compare M-CBT to a CBT group therapy consisting primarily of cognitive restructuring. It cannot be determined whether the treatment effects were enhanced by the combination of MT with cognitive restructuring. Future research may benefit from comparing the effects of a program consisting primarily of cognitive restructuring and the four-session M-CBT program to clarify whether the addition of MT to cognitive restructuring enhances the effects on cost/probability bias and social anxiety symptoms. Validation of the effectiveness of M-CBT through rigorous RCTs that address the limitations of this study may provide evidence supporting an additional treatment module for patients who have not shown a therapeutic response to conventional CBT, particularly regarding cost/probability bias and social anxiety symptoms.

Further, the participants were Japanese university students. Since the psychopathology of social anxiety differs across cultures [72], it is suggested that intervention programs may also differ in effectiveness across countries. Therefore, there is a need to examine the generalizability of M-CBT to other populations and cultures.

## Conclusions

This study showed that M-CBT was effective in the treatment of negative cognition generated when paying attention to others in probability bias, fear of negative evaluation by others, dispositional mindfulness, depressive symptoms, and subjective happiness. This study provides an impetus for future research on the combination of MT with cognitive–behavioral techniques for treating social anxiety symptoms and cost/probability bias. Rigorous RCTs that address the study limitations should be conducted to further examine the utility of M-CBT.

### Supplementary Information

**Supplementary Material 1.**

**Supplementary Material 2.**

**Supplementary Material 3.**

**Supplementary Material 4.**

## Data Availability

Detailed data are available from the corresponding authors upon reasonable request.
