# Supplementary material for: Low-intensity mindfulness and cognitive–behavioral therapy for social anxiety: a pilot randomized controlled trial
Source: BMC Psychiatry. 2024 Mar 7;24:190. doi: 10.1186/s12888-024-05651-0 (PMC10921717; doi:10.1186/s12888-024-05651-0)
Supplement: Supplementary file 1 — Supplementary Material 1. [file 12888_2024_5651_MOESM1_ESM.docx]

MCBT protocol

The four-session program of mindfulness and cognitive behavioral therapy (M-CBT) consisted mainly of psychoeducation, MT, cognitive restructuring, and sharing to decrease cost/probability bias and social anxiety symptoms. In the unified protocol for transdiagnostic treatment of emotional disorders, which adds an MT component to the CBT protocol, the first half of all sessions is built primarily on psychoeducation, self-monitoring, MT, and cognitive restructuring, while the second half is built primarily on exposure therapy for internalized sensations and emotions (1). Because the M-CBT program consists primarily of psychoeducation, MT, and cognitive restructuring, our program, designed as a brief, low-intensity treatment module, consisted of a total of four sessions—half the number of sessions required by an MBI.

The Purpose of M-CBT

The aims of the four-session M-CBT program are discussed below.

1. Awareness of one’s reaction patterns in social situations

Heimberg (2) indicated that an awareness of one’s thoughts in anxiety-provoking situations is important in cognitive restructuring. MT promotes awareness of response patterns, including thoughts, emotions, and body sensations (3). Accordingly, the M-CBT program aimed to increase awareness of one’s response patterns using MT. Participants observed their reaction patterns to fearful social situations. They deepened their self-monitoring in social situations by noticing their negative thinking habits, emotions such as anxiety and fear, physical sensations such as trembling, sensitive reactions to fearful things, and self-focused attention on their performance.

1. Acceptance of one’s reaction patterns in social situations

Patients with mental illnesses are often preoccupied with negative cognitions (4). This is reduced by paying attention to the present moment and accepting the present experience in a non-judgmental manner (3). Therefore, the M-CBT program aimed to help individuals accept their response pattern using MT and realize that their present thoughts did not define them. The participants attempted to acquire the attitude of self-acceptance.

1. Letting go of one’s negative thoughts

Beck et al. (4) suggested that cognitive restructuring is essential to distance oneself from one’s thoughts and regard them as psychological events rather than reality. By distancing themselves, individuals can acquire a more realistic and adaptive way of thinking. MT increases awareness and acceptance of one’s negative perceptions and enhances the skill of distancing oneself from one’s thoughts (5, 6). Therefore, through MT, the M-CBT program aimed to impart the skills to let go of thoughts that arise in social situations. The participants learned how to accept that negative thoughts are their own making and not necessarily true, and to wait for them to fade away instead of suppressing them.

1. Considering things from an objective perspective

Heimberg (2) indicated that constructive alternative thinking is derived from an objective perspective in cognitive restructuring. The M-CBT program aimed to enhance the ability to take an objective view of things. Through MT, the participants attempted to improve their awareness of their reaction patterns and distance themselves from their thoughts. Through cognitive restructuring, participants improved their ability to view things objectively. Participants also acquired the skills to find constructive thoughts to fit a situation. They could realize the subjectivity of their perception when negative thoughts arose and attempted to adopt an objective perspective.

The Four-Session Program

Session 1: Discover the Factors That Are Increasing Social Anxiety

In Session 1, mindful yoga was first performed to relieve tension in the group. Thereafter, the treatment plan was explained to the participants, and they engaged in a motivational exercise in which treatment goals and expectations were discussed. Participants learned about social anxiety and mindfulness through psychoeducation. They were briefed on the mechanisms of maintaining social anxiety, particularly the role of negative thoughts in increasing social anxiety. They were taught the three types of negative cognition: rumination, cost bias, and probability bias, and derived an individual personal model using these definitions. Subsequently, the concept of mindfulness and how it helps reduce negative thoughts were explained. After the psychoeducation, sitting meditation (about 25 minutes) with a focus on breathing and one’s thoughts was conducted. Through MT, participants were expected to become aware of their current thoughts. Finally, participants shared with group members what they had discovered about themselves through the session.

Session 2: Identify the Factors That Are Causing Social Anxiety

In Session 2, mindful yoga was performed to relieve tension in the group. Participants learned more about social anxiety and mindfulness through psychoeducation. They were told about the mental responses to anxiety-provoking situations, including thoughts and emotions, physical sensations, and avoidance behaviors. The purpose of Session 2 was to identify the factors that were causing one’s social anxiety, and we attempted to achieve this goal through a mindfulness practice. In the mindfulness practice (about 40 minutes), the participants performed sitting meditation, during which, they imagined anxiety-provoking situations and tried to notice their response patterns. They tried to identify the negative thoughts that increased their social anxiety and become aware of their response patterns using a cognitive-behavioral model that included thoughts (rumination, cost bias, and probability bias), emotions, physical sensations, and avoidance behaviors. Finally, they shared their learning through the session.

Session 3: Observe the Factors That Are Causing Social Anxiety

Session 3 focused on mindfulness practices to confront and let go of negative thoughts that increase social anxiety. The participants made an anxiety hierarchy list and identified anxiety-provoking situations. In addition, they created the cognitive-behavioral model comprising four variables (thoughts, emotions, physical sensations, and avoidance behaviors). In the mindfulness practice (about 40 minutes), participants imagined anxiety-provoking situations and attempted to notice their negative thoughts. They did not criticize or suppress the thoughts but observed them and experienced the process of waiting for them to disappear. After the mindfulness practice, the cognitive-behavioral model was created again. Subsequently, the experience of awareness and letting go of thoughts achieved through mindfulness practice was shared.

Session 4: Let Go of the Factors That Are Causing Social Anxiety

Session 4 was designed to help participants acquire the skills to think objectively rather than subjectively. Mindfulness practice (about 20 minutes) using imagery of anxiety-provoking situations was conducted to help participants notice their negative thoughts. Cognitive restructuring using a thought record was implemented, which included the following three stages:

1. noticing one’s thoughts and feelings,
2. reflecting on the appropriateness of the thoughts from an objective perspective, and
3. finding constructive thoughts based on the information obtained from the objective perspective.

The participants applied the skill of viewing things from different perspectives instead of just subjective ones. Specifically, participants gained skills to acquire realistic and constructive thoughts while accepting the negative ones that arise from taking a subjective view of things. Hofmann et al. (7) and Hofmann et al. (8) suggested that loving-kindness meditation is effective for positive and negative emotions and may provide potentially useful strategies for targeting social anxiety by combining CBT. Thus, loving-kindness meditation (about 15 minutes) was included in this session. Finally, participants shared their learning throughout the program.

**Homework**

Participants were assigned homework to incorporate the practices from each session into their daily lives. Homework for Session 1 included sitting meditation and a diary of daily happiness. Participants were given a meditation guide that included an audio recording of guided sitting meditation and were instructed to meditate for at least 20 minutes each day at home. The diary of daily happiness is intended to help participants become aware of instances of small happiness in their daily lives. Participants were asked to describe pleasant events, their thoughts, feelings, and physical sensations at the time, and what thoughts emerge when they look back on the events. Homework for Session 2 included sitting or imagery meditation and a diary of thoughts, emotions, behaviors, and physical reactions in interaction situations with others. The goal of the audio recording with guided imagery meditation given to participants was to help them become aware of how they react in an anxiety-provoking situation. The diary of thoughts, feelings, behaviors, and physical reactions in interaction situations with others is intended to help participants become aware of their responses in interaction situations with others. Participants were asked to describe events related to interacting with others, their thoughts, feelings, and physical sensations at the time, and what thoughts emerge when they reflect on the events. Homework for Session 3 included sitting or imagery meditation and a diary of communication with others. Participants received an audio recording with guided imagery meditation, which aimed to observe their responses in anxiety-provoking situations and experience the process of waiting for them to pass. Participants were asked to describe events in which they communicated with others, their thoughts, feelings, physical sensations, and behaviors at the time, what makes communicating with others difficult, and what they have learned from that communication. Homework for Session 4 was to continue to meditate for their own mental health.

**Place and Therapist**

The M-CBT was conducted at the university in Tokyo. Participants could freely borrow a yoga mat and zafu (cushion), and materials were distributed for free. The program was conducted using PowerPoint. A clinical psychologist with more than two years of clinical experience practicing psychotherapy in clinical settings and more than two years of experience in mindfulness practice led all of the group sessions. For CBT, the therapist was supervised by a clinical psychologist with more than 10 years of clinical experience, and for MT, by a mindfulness-based stress reduction teacher with more than five years of clinical experience.

References

1. Barlow DH, Green JD, Fairholme CP, Elland KK, Boisseau CL, Allen LB, & Ehrenreich-May JT. Unified protocol for transdiagnostic treatment of emotional disorder: Therapist guide. Oxford University Press;2011.
2. Heimberg RG. Cognitive-behavioral therapy for social anxiety disorder: current status and future directions. Biol Psychiatry. 2002;51(1):101-8. <https://doi.org/10.1016/s0006-3223(01)01183-0>
3. Kabat-Zinn J. Wherever you go, there you are: Mindfulness meditation in everyday life. Hyperion;1994.
4. Beck AT, Rush AJ, Shaw BF, Emery G. Cognitive therapy of depression. Guilford Press;1979.
5. Segal ZV, Williams JM, Teasdale J. Mindfulness-based cognitive therapy for depression. Guilford Press;2002.
6. Carmody J, Baer RA, Lykins ELB, Olendzki N. An empirical study of the mechanisms of mindfulness in a mindfulness-based stress reduction program. J Clin Psychol. 2009;65(6):613-26. <https://doi.org/10.1002/jclp.20579>
7. Hofmann SG, Grossman P, Hinton DE. Loving-kindness and compassion meditation: potential for psychological interventions. Clin Psychol Rev. 2011;31(7):1126-32. <https://doi.org/10.1016/j.cpr.2011.07.003>
8. Hofmann SG, Petrocchi N, Steinberg J, Lin M, Arimitsu K, Kind S, Mendes A, Stangier U. Loving-kindness meditation to target affect in mood disorders: A proof-of-concept study. Evid Based Complement Alternat Med. 2015;269126. <https://doi.org/10.1155/2015/269126>
