# Supplementary material for: Low-intensity mindfulness and cognitive–behavioral therapy for social anxiety: a pilot randomized controlled trial
Source: BMC Psychiatry. 2024 Mar 7;24:190. doi: 10.1186/s12888-024-05651-0 (PMC10921717; doi:10.1186/s12888-024-05651-0)
Supplement: Supplementary file 2 — Supplementary Material 2. [file 12888_2024_5651_MOESM2_ESM.docx]

**Table S1**

*Results of Kolmogorov-Smirnov Normality Test for intent-to-treat analysis.*

|  |  | **Intervention group** | | |  | **Control group** | | |
| --- | --- | --- | --- | --- | --- | --- | --- | --- |
|  |  | Statistic | df | *p*-value |  | Statistic | df | *p*-value |
| **Primary outcomes** |  |  |  |  |  |  |  |  |
| LSAS total score | Pre-test | .10 | 27 | > .200 |  | .18 | 23 | .06 |
|  | Post-test | .12 | 27 | > .200 |  | .07 | 23 | > .200 |
|  | Follow-up | .12 | 27 | > .200 |  | .10 | 23 | > .200 |
|  |  |  |  |  |  |  |  |  |
| LSAS anxiety | Pre-test | .14 | 27 | .18 |  | .15 | 23 | > .200 |
|  | Post-test | .12 | 27 | > .200 |  | .12 | 23 | > .200 |
|  | Follow-up | .13 | 27 | > .200 |  | .11 | 23 | > .200 |
|  |  |  |  |  |  |  |  |  |
| LSAS avoidance behavior | Pre-test | .09 | 27 | > .200 |  | .14 | 23 | > .200 |
|  | Post-test | .13 | 27 | > .200 |  | .12 | 23 | > .200 |
|  | Follow-up | .09 | 27 | > .200 |  | .10 | 23 | > .200 |
|  |  |  |  |  |  |  |  |  |
| SCPS cost bias total score | Pre-test | .11 | 27 | > .200 |  | .17 | 23 | .09 |
|  | Post-test | .10 | 27 | > .200 |  | .14 | 23 | > .200 |
|  | Follow-up | .11 | 27 | > .200 |  | .19 | 23 | <.05 |
|  |  |  |  |  |  |  |  |  |
| SCPS cost bias Negative cognition from one's performance | Pre-test | .10 | 27 | > .200 |  | .14 | 23 | > .200 |
|  | Post-test | .08 | 27 | > .200 |  | .14 | 23 | > .200 |
|  | Follow-up | .11 | 27 | > .200 |  | .16 | 23 | .13 |
|  |  |  |  |  |  |  |  |  |
| SCPS cost bias Negative cognition generated when paying attention to others | Pre-test | .14 | 27 | .16 |  | .11 | 23 | > .200 |
|  | Post-test | .16 | 27 | .08 |  | .13 | 23 | > .200 |
|  | Follow-up | .15 | 27 | .11 |  | .20 | 23 | <.05 |
|  |  |  |  |  |  |  |  |  |
| SCPS probability bias total score | Pre-test | .12 | 27 | > .200 |  | .09 | 23 | > .200 |
|  | Post-test | .18 | 27 | <.05 |  | .11 | 23 | > .200 |
|  | Follow-up | .18 | 27 | <.05 |  | .10 | 23 | > .200 |
|  |  |  |  |  |  |  |  |  |
| SCPS probability bias Negative cognition from one's performance | Pre-test | .10 | 27 | > .200 |  | .12 | 23 | > .200 |
|  | Post-test | .21 | 27 | <.01 |  | .09 | 23 | > .200 |
|  | Follow-up | .17 | 27 | <.05 |  | .13 | 23 | > .200 |
|  |  |  |  |  |  |  |  |  |
| SCPS probability bias Negative cognition generated when paying attention to others | Pre-test | .16 | 27 | .07 |  | .10 | 23 | > .200 |
|  | Post-test | .12 | 27 | > .200 |  | .14 | 23 | > .200 |
|  | Follow-up | .22 | 27 | <.01 |  | .21 | 23 | <.05 |
|  |  |  |  |  |  |  |  |  |
| **Secondary outcomes** |  |  |  |  |  |  |  |  |
| SFA total score | Pre-test | .12 | 27 | > .200 |  | .14 | 23 | > .200 |
|  | Post-test | .09 | 27 | > .200 |  | .13 | 23 | > .200 |
|  | Follow-up | .14 | 27 | > .200 |  | .15 | 23 | > .200 |
|  |  |  |  |  |  |  |  |  |
| SFA arousal | Pre-test | .13 | 27 | > .200 |  | .17 | 23 | .09 |
|  | Post-test | .12 | 27 | > .200 |  | .11 | 23 | > .200 |
|  | Follow-up | .11 | 27 | > .200 |  | .15 | 23 | > .200 |
|  |  |  |  |  |  |  |  |  |
| SFA behavior | Pre-test | .17 | 27 | <.05 |  | .13 | 23 | > .200 |
|  | Post-test | .17 | 27 | .06 |  | .17 | 23 | .09 |
|  | Follow-up | .16 | 27 | .06 |  | .15 | 23 | .19 |
|  |  |  |  |  |  |  |  |  |
| SFNE total score | Pre-test | .09 | 27 | > .200 |  | .16 | 23 | .16 |
|  | Post-test | .19 | 27 | .02 |  | .18 | 23 | .06 |
|  | Follow-up | .07 | 27 | > .200 |  | .17 | 23 | .08 |
|  |  |  |  |  |  |  |  |  |
| SFNE forward-item | Pre-test | .10 | 27 | > .200 |  | .13 | 23 | > .200 |
|  | Post-test | .21 | 27 | <.01 |  | .22 | 23 | <.01 |
|  | Follow-up | .09 | 27 | > .200 |  | .13 | 23 | > .200 |
|  |  |  |  |  |  |  |  |  |
| SFNE reversed-item | Pre-test | .13 | 27 | > .200 |  | .22 | 23 | <.01 |
|  | Post-test | .11 | 27 | > .200 |  | .24 | 23 | <.01 |
|  | Follow-up | .13 | 27 | > .200 |  | .17 | 23 | .10 |
|  |  |  |  |  |  |  |  |  |
| **Additional outcomes** |  |  |  |  |  |  |  |  |
| FFMQ | Pre-test | .11 | 27 | > .200 |  | .17 | 23 | .08 |
|  | Post-test | .16 | 27 | .06 |  | .12 | 23 | > .200 |
|  | Follow-up | .08 | 27 | > .200 |  | .10 | 23 | > .200 |
|  |  |  |  |  |  |  |  |  |
| SDS | Pre-test | .11 | 27 | > .200 |  | .11 | 23 | > .200 |
|  | Post-test | .13 | 27 | > .200 |  | .14 | 23 | > .200 |
|  | Follow-up | .08 | 27 | > .200 |  | .09 | 23 | > .200 |
|  |  |  |  |  |  |  |  |  |
| SHS | Pre-test | .12 | 27 | > .200 |  | .20 | 23 | <.05 |
|  | Post-test | .11 | 27 | > .200 |  | .15 | 23 | > .200 |
|  | Follow-up | .13 | 27 | > .200 |  | .10 | 23 | > .200 |
|  |  |  |  |  |  |  |  |  |

*Note.* FFMQ = Five Facet Mindfulness Questionnaire; SCPS = Speech Cost/Probability Bias Scale; SFNE = Short Fear of Negative Evaluation Scale; SDS = Self-rating Depression Scale; SFA = Self-focused Attention Scale; SHS = Subjective Happiness Scale; LSAS = Liebowitz Social Anxiety Scale

**Table S2**

*Results of Kolmogorov-Smirnov Normality Test for per-protocol analysis.*

|  |  | **Intervention group** | | |  | **Control group** | | |
| --- | --- | --- | --- | --- | --- | --- | --- | --- |
|  |  | Statistic | df | *p*-value |  | Statistic | df | *p*-value |
| **Primary outcomes** |  |  |  |  |  |  |  |  |
| LSAS total score | Pre-test | .11 | 26 | > .200 |  | .18 | 23 | .06 |
|  | Post-test | .12 | 26 | > .200 |  | .07 | 23 | > .200 |
|  | Follow-up | .13 | 26 | > .200 |  | .11 | 22 | > .200 |
|  |  |  |  |  |  |  |  |  |
| LSAS anxiety | Pre-test | .15 | 26 | .11 |  | .15 | 23 | > .200 |
|  | Post-test | .13 | 26 | > .200 |  | .12 | 23 | > .200 |
|  | Follow-up | .14 | 26 | > .200 |  | .13 | 22 | > .200 |
|  |  |  |  |  |  |  |  |  |
| LSAS avoidance behavior | Pre-test | .09 | 26 | > .200 |  | .14 | 23 | > .200 |
|  | Post-test | .14 | 26 | > .200 |  | .12 | 23 | > .200 |
|  | Follow-up | .11 | 26 | > .200 |  | .09 | 22 | > .200 |
|  |  |  |  |  |  |  |  |  |
| SCPS cost bias total score | Pre-test | .12 | 26 | > .200 |  | .17 | 23 | .09 |
|  | Post-test | .11 | 26 | > .200 |  | .14 | 23 | > .200 |
|  | Follow-up | .12 | 26 | > .200 |  | .18 | 22 | .07 |
|  |  |  |  |  |  |  |  |  |
| SCPS cost bias Negative cognition from one's performance | Pre-test | .10 | 26 | > .200 |  | .14 | 23 | > .200 |
|  | Post-test | .08 | 26 | > .200 |  | .14 | 23 | > .200 |
|  | Follow-up | .11 | 26 | > .200 |  | .15 | 22 | .20 |
|  |  |  |  |  |  |  |  |  |
| SCPS cost bias Negative cognition generated when paying attention to others | Pre-test | .16 | 26 | .08 |  | .11 | 23 | > .200 |
|  | Post-test | .16 | 26 | .07 |  | .13 | 23 | > .200 |
|  | Follow-up | .14 | 26 | .18 |  | .18 | 22 | .07 |
|  |  |  |  |  |  |  |  |  |
| SCPS probability bias total score | Pre-test | .12 | 26 | > .200 |  | .09 | 23 | > .200 |
|  | Post-test | .17 | 26 | .05 |  | .11 | 23 | > .200 |
|  | Follow-up | .18 | 26 | <.05 |  | .11 | 22 | > .200 |
|  |  |  |  |  |  |  |  |  |
| SCPS probability bias Negative cognition from one's performance | Pre-test | .11 | 26 | > .200 |  | .12 | 23 | > .200 |
|  | Post-test | .20 | 26 | <.05 |  | .09 | 23 | > .200 |
|  | Follow-up | .17 | 26 | .06 |  | .13 | 22 | > .200 |
|  |  |  |  |  |  |  |  |  |
| SCPS probability bias Negative cognition generated when paying attention to others | Pre-test | .15 | 26 | .15 |  | .10 | 23 | > .200 |
|  | Post-test | .13 | 26 | > .200 |  | .14 | 23 | > .200 |
|  | Follow-up | .23 | 26 | <.01 |  | .20 | 22 | <.05 |
|  |  |  |  |  |  |  |  |  |
| **Secondary outcomes** |  |  |  |  |  |  |  |  |
| SFA total score | Pre-test | .12 | 26 | > .200 |  | .14 | 23 | > .200 |
|  | Post-test | .07 | 26 | > .200 |  | .13 | 23 | > .200 |
|  | Follow-up | .12 | 26 | > .200 |  | .14 | 22 | > .200 |
|  |  |  |  |  |  |  |  |  |
| SFA arousal | Pre-test | .14 | 26 | .19 |  | .17 | 23 | .09 |
|  | Post-test | .13 | 26 | > .200 |  | .11 | 23 | > .200 |
|  | Follow-up | .11 | 26 | > .200 |  | .13 | 22 | > .200 |
|  |  |  |  |  |  |  |  |  |
| SFA behavior | Pre-test | .17 | 26 | .06 |  | .13 | 23 | > .200 |
|  | Post-test | .16 | 26 | .07 |  | .17 | 23 | .09 |
|  | Follow-up | .17 | 26 | .06 |  | .14 | 22 | > .200 |
|  |  |  |  |  |  |  |  |  |
| SFNE total score | Pre-test | .10 | 26 | > .200 |  | .16 | 23 | .16 |
|  | Post-test | .21 | 26 | <.01 |  | .18 | 23 | .06 |
|  | Follow-up | .10 | 26 | > .200 |  | .16 | 22 | .17 |
|  |  |  |  |  |  |  |  |  |
| SFNE forward-item | Pre-test | .09 | 26 | > .200 |  | .13 | 23 | > .200 |
|  | Post-test | .23 | 26 | <.01 |  | .22 | 23 | <.01 |
|  | Follow-up | .12 | 26 | > .200 |  | .12 | 22 | > .200 |
|  |  |  |  |  |  |  |  |  |
| SFNE reversed-item | Pre-test | .14 | 26 | > .200 |  | .22 | 23 | <.01 |
|  | Post-test | .11 | 26 | > .200 |  | .24 | 23 | <.01 |
|  | Follow-up | .14 | 26 | > .200 |  | .17 | 22 | .08 |
|  |  |  |  |  |  |  |  |  |
| **Additional outcomes** |  |  |  |  |  |  |  |  |
| FFMQ | Pre-test | .13 | 26 | > .200 |  | .17 | 23 | .08 |
|  | Post-test | .18 | 26 | <.05 |  | .12 | 23 | > .200 |
|  | Follow-up | .10 | 26 | > .200 |  | .10 | 22 | > .200 |
|  |  |  |  |  |  |  |  |  |
| SDS | Pre-test | .10 | 26 | > .200 |  | .11 | 23 | > .200 |
|  | Post-test | .11 | 26 | > .200 |  | .14 | 23 | > .200 |
|  | Follow-up | .09 | 26 | > .200 |  | .09 | 22 | > .200 |
|  |  |  |  |  |  |  |  |  |
| SHS | Pre-test | .13 | 26 | > .200 |  | .20 | 23 | <.05 |
|  | Post-test | .11 | 26 | > .200 |  | .15 | 23 | > .200 |
|  | Follow-up | .13 | 26 | > .200 |  | .11 | 22 | > .200 |
|  |  |  |  |  |  |  |  |  |

*Note.* FFMQ = Five Facet Mindfulness Questionnaire; SCPS = Speech Cost/Probability Bias Scale; SFNE = Short Fear of Negative Evaluation Scale; SDS = Self-rating Depression Scale; SFA = Self-focused Attention Scale; SHS = Subjective Happiness Scale; LSAS = Liebowitz Social Anxiety Scale
