# Supplementary material for: Low-intensity mindfulness and cognitive–behavioral therapy for social anxiety: a pilot randomized controlled trial
Source: BMC Psychiatry. 2024 Mar 7;24:190. doi: 10.1186/s12888-024-05651-0 (PMC10921717; doi:10.1186/s12888-024-05651-0)
Supplement: Supplementary file 4 — Supplementary Material 4. [file 12888_2024_5651_MOESM4_ESM.docx]

Per-Protocol Analysis Sample

*Primary outcomes*

The results of the group × time (2 × 2) ANCOVA showed significant interactions in the SCPS Cost bias in the negative cognition generated when paying attention to others: *F*(1, 46) = 4.09, *p* < .05; SCPS Probability bias total score: *F*(1, 46) = 11.11, *p* < .01; SCPS Probability bias in the negative cognition from one’s performance: *F*(1, 46) = 4.71, *p* < .05; SCPS Probability bias in the negative cognition generated when paying attention to others: *F*(1, 46) = 21.95, *p* < .01. Simple effect analyses using Bonferroni’s method for these variables showed significant differences. For these variables, the post-test revealed significantly lower scores than the pre-test in the intervention group (*p* < .05). At the post-test, the intervention group had significantly lower scores than the control group for these variables (*p* < .01). Conversely, no interactions were observed in the LSAS total score: *F*(1, 46) = .33, *p* = .57, anxiety: *F*(1, 46) = .21, *p* = .65, and avoidance behavior: *F*(1, 46) = .51，*p* = .48; the SCPS Cost bias total score: *F*(1, 46) = .50, *p* = .48; SCPS Cost bias in the negative cognition from one’s performance: *F*(1, 46) = .00, *p* = .98. Significant main effects were found between the pre-test and post-test within participants in the LSAS total score, avoidance behavior, SCPS Cost bias total score, and SCPS Cost bias in the negative cognition from one’s performance (*p* < .05), except for anxiety (*p* = .10). Multiple comparisons using Bonferroni’s method were performed for the LSAS total score, avoidance behavior, SCPS Cost bias total score, and SCPS Cost bias in the negative cognition from one’s performance. In the LSAS, and avoidance behavior, the post-test had significantly lower scores than the pre-test (*p* < .05). No significant main effect was obtained in the LSAS total score (*p* = .57), anxiety (*p* = .65), avoidance behavior (*p* = .48), SCPS Cost bias total score (*p* = .48), and SCPS Cost bias in the negative cognition from one’s performance (*p* = .98).

The results of the group × time (2 × 3) ANCOVA showed significant interactions in the SCPS Probability bias total score: *F*(2, 90)=4.27, *p* <.05; SCPS Probability bias in the negative cognition generated when paying attention to others: *F*(2, 90) = 7.94, *p* < .01. Simple effect analyses using Bonferroni’s method for these variables showed significant differences. For these variables, the post-test and follow-up had significantly lower scores than the pre-test in the intervention group (*p* < .05). At the post-test and follow-up, the intervention group had significantly lower scores than the control group in the Probability bias in the negative cognition generated when paying attention to others and the intervention group had significantly lower scores than the control group in the SCPS Probability bias total score at the post-test (*p* < .01). However, there were no interactions in the LSAS total score: *F*(2, 90) = .64, *p* = .53, anxiety: *F*(2, 90) = .39, *p* = .68, and avoidance behavior: *F*(2, 90) = .82, *p* = .44; the SCPS Cost bias total sore: *F*(2, 90) = 1.88, *p* = .16; SCPS Cost bias in the negative cognition from one’s performance: *F*(2, 90) = 1.52, *p* = .23; SCPS Cost bias in the negative cognition generated when paying attention to others: *F*(2, 90) = 2.57, p = .08; SCPS Probability bias in the negative cognition from one’s performance: *F*(2, 90) = 2.09, *p* = .13. Significant main effects were acquired between the pre-test, post-test, and follow-up within participants in avoidance behavior, SCPS Cost bias total score, SCPS Cost bias in the negative cognition from one’s performance, SCPS Cost bias in the negative cognition generated when paying attention to others, and SCPS Probability bias in the negative cognition from one’s performance (*p* < .05), except for the LSAS total score (*p* = .05) and anxiety (*p* = .15). Multiple comparisons using Bonferroni’s method were performed for avoidance behavior, the SCPS Cost bias total score, SCPS Cost bias in the negative cognition from one’s performance, SCPS Cost bias in the negative cognition generated when paying attention to others, and SCPS Probability bias in the negative cognition from one’s performance. In the SCPS Cost bias in the negative cognition generated when paying attention to others and SCPS Probability bias in the negative cognition from one’s performance, the post-test and follow-up had significantly lower scores than the pre-test (*p* < .05). In the SCPS Cost bias total score and SCPS Cost bias in the negative cognition from one’s performance, the post-test had significantly lower scores than the pre-test (*p* < .05). A significant main effect was observed between the intervention and control groups in the SCPS Cost bias in the negative cognition generated when paying attention to others; however, no significant main effect was found in the LSAS total score (*p* = .39), anxiety (*p* = .49), avoidance behavior (*p* = .30), SCPS Cost bias total score (*p* = .16), SCPS Cost bias in the negative cognition from one’s performance (*p* = .44), and SCPS Probability bias in the negative cognition from one’s performance (*p* = .16). Multiple comparisons using Bonferroni’s method were performed for the SCPS Cost bias in the negative cognition generated when paying attention to others; the intervention group had significantly higher scores than the control group (*p* < .05).

*Secondary outcomes*

The results of the group × time (2 × 2) ANCOVA showed significant interactions in the SFNE total score: *F*(1, 46) = 7.39, *p* < .01; SFNE forward-item: *F*(1, 46) = 7.58, *p* < .01; SFNE reversed-item: *F*(1, 46) = 5.24, *p* < .05. Simple effect analyses using Bonferroni’s method for these variables showed significant differences. For these variables, the post-test had significantly lower scores than the pre-test in the intervention group (*p* < .01). At the post-test, the intervention group had significantly lower scores than the control group for these variables (*p* < .05). Conversely, no interactions were evident in the SFA total score: *F*(1, 46) = .19, *p* = .67; SFA arousal: *F*(1, 46) = .10, *p* = .75; SFA behavior: *F*(1, 46) = 1.90, *p* = .18. Significant main effects were observed between the pre-test and post-test within participants for these variables (*p* < .05). Multiple comparisons using Bonferroni’s method were performed for these variables between the pre-test and post-test; the post-test had significantly lower scores than the pre-test in the SFA total score (*p* < .05). No significant main effect was found between the intervention and control groups in the SFA total score (*p* = .67), SFA arousal (*p* = .75), and SFA behavior (*p* = .18).

The results of the group × time (2 × 3) ANCOVA showed significant interactions in the SFNE total score: *F*(1.59, 71.55) = 9.57, *p* < .01; SFNE forward-item: *F*(1.72, 77.44) = 8.77, *p* < .01; SFNE forward-item: *F*(1.71, 76.79) = 5.81, *p* < .01. Simple effect analyses using Bonferroni’s method for these variables showed significant differences. For these variables, the post-test and follow-up had significantly lower scores than the pre-test in the intervention group (*p* < .05). At the post-test and follow-up, the intervention group had significantly lower scores than the control group for these variables (*p* < .05). However, no interactions were found in the SFA total score: *F*(2, 90) = .35, *p* = .71; SFA arousal: *F*(2, 90) = .23, *p* = .80; SFA behavior: *F*(2, 90) = 1.30, *p* = .28. Significant main effects were observed between the pre-test, post-test, and follow-up within participants in the SFA total score and SFA behavior (*p* < .05), except for the SFA arousal (*p* = .10). Multiple comparisons using Bonferroni’s method were performed for the SFA total score and SFA behavior between the pre-test, post-test, and follow-up; the follow-up had significantly lower scores than the pre-test in the SFA total score and SFA behavior (*p* < .05). No significant main effect was evident between the intervention and control groups in the SFA total score (*p* = .54), SFA arousal (*p* = .93), and SFA behavior (*p* = .09).

***Additional outcomes***

The results of the group × time (2 × 2) ANCOVA showed significant interactions in the FFMQ: *F*(1, 46) = 39.54, *p* < .01; SDS: *F*(1, 46) = 14.61, *p* < .01; and SHS: *F*(1, 46) = 20.11, *p* < .01. Simple effect analyses using Bonferroni’s method for these variables showed significant differences. In the FFMQ and SHS, the post-test had significantly higher scores than the pre-test in the intervention group (*p* < .01) and the post-test had significantly lower scores than the pre-test in the SDS (*p* < .01). At the post-test, the intervention group had significantly higher scores than the control group in the FFMQ and SHS and the intervention group had significantly lower scores than the control group in the SDS (*p* < .01).

The results of the group × time (2 × 3) ANCOVA showed significant interactions in the FFMQ: *F*(1.55, 69.70) = 24.16, *p* < .01; SDS: *F*(1.76, 79.31) = 11.92, *p* < .01; and SHS: *F*(1.49, 67.04) = 14.34, *p* < .01. Simple effect analyses using Bonferroni’s method for these variables showed significant differences. In the FFMQ and SHS, the post-test and follow-up had significantly higher scores than the pre-test in the intervention group (*p* < .01) and the post-test and follow-up had significantly lower scores than the pre-test in the SDS (*p* < .01). At the post-test and follow-up, the intervention group had significantly higher scores than the control group in the FFMQ and SHS and the intervention group had significantly lower scores than the control group in the SDS (*p* < .01).

**Figure S1**

*Changes in the total score of the Liebowitz Social Anxiety Scale in the per-protocol analysis.*


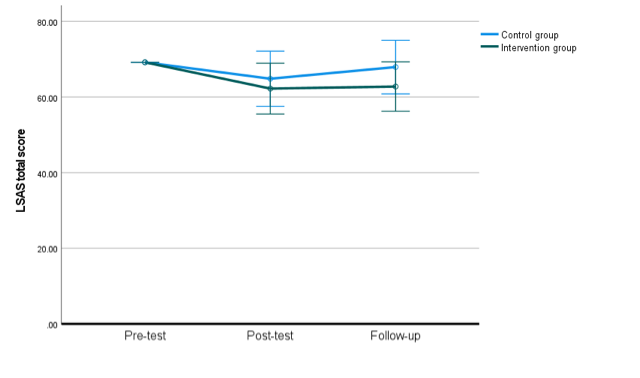


*Note.* Covariates appearing in the model are evaluated at Pre-test = 69.17; Error bars = 95% confidence interval; LSAS = Liebowitz Social Anxiety Scale.

**Figure S2**

*Changes in the anxiety score of the Liebowitz Social Anxiety Scale in the per-protocol analysis.*


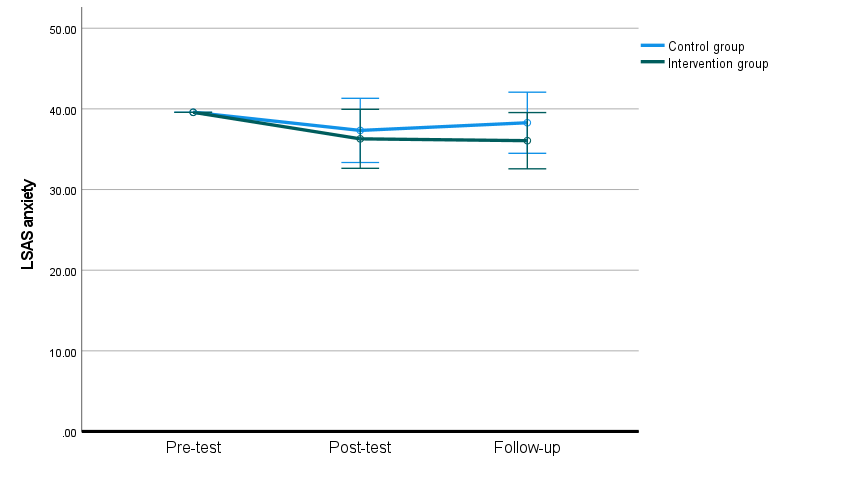


*Note.* Covariates appearing in the model are evaluated at Pre-test = 39.58; Error bars = 95% confidence interval; LSAS = Liebowitz Social Anxiety Scale.

**Figure S3**

*Changes in the avoidance behavior score of the Liebowitz Social Anxiety Scale in the per-protocol analysis.*


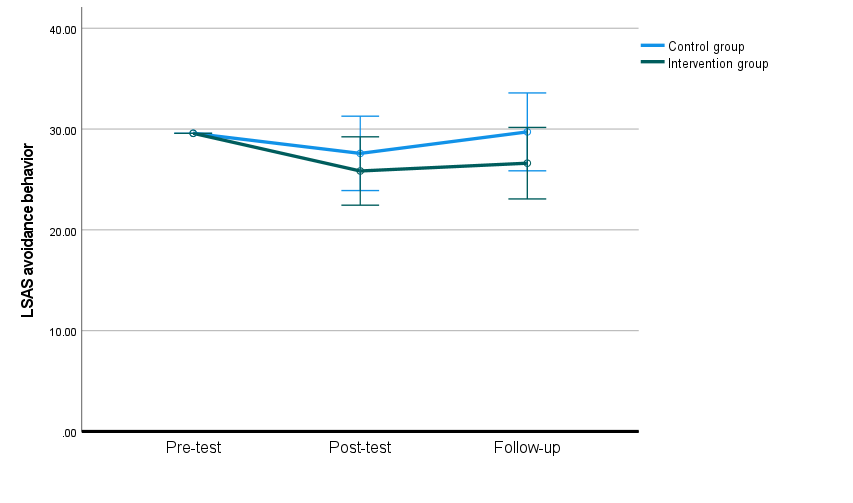


*Note.* Covariates appearing in the model are evaluated at Pre-test = 29.58; Error bars = 95% confidence interval; LSAS = Liebowitz Social Anxiety Scale.

**Figure S4**

*Changes in the total score of the Speech Cost/Probability bias Scale cost bias in the per-protocol analysis.*


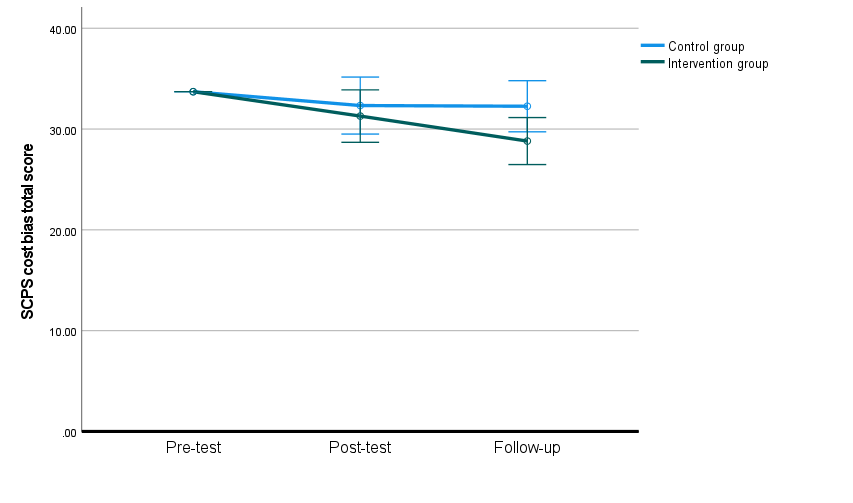


*Note.* Covariates appearing in the model are evaluated at Pre-test = 33.71; Error bars = 95% confidence interval; SCPS = Speech Cost/Probability bias Scale.

**Figure S5**

*Changes in the negative cognition from one’s performance score of the Speech Cost/Probability bias Scale cost bias in the per-protocol analysis.*


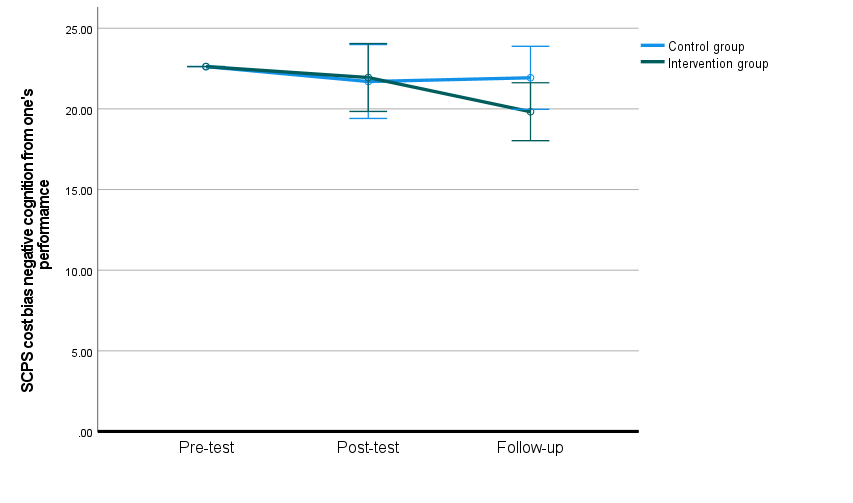


*Note.* Covariates appearing in the model are evaluated at Pre-test = 22.63; Error bars = 95% confidence interval; SCPS = Speech Cost/Probability bias Scale.

**Figure S6**

*Changes in the negative cognition generated when paying attention to others score of the Speech Cost/Probability bias Scale cost bias in the per-protocol analysis.*


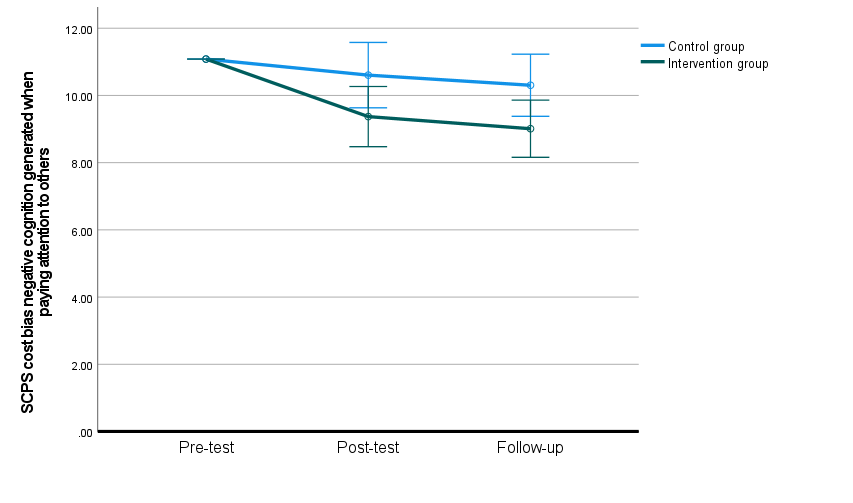


*Note.* Covariates appearing in the model are evaluated at Pre-test = 11.08; Error bars = 95% confidence interval; SCPS = Speech Cost/Probability bias Scale.

**Figure S7**

*Changes in the total score of the Speech Cost/Probability bias Scale probability bias in the per-protocol analysis.*


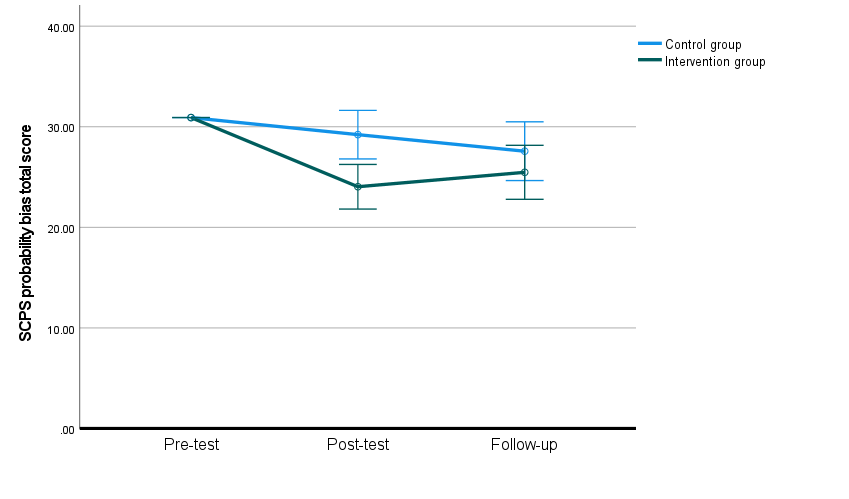


*Note.* Covariates appearing in the model are evaluated at Pre-test = 30.92; Error bars = 95% confidence interval; SCPS = Speech Cost/Probability bias Scale.

**Figure S8**

*Changes in the negative cognition from one’s performance score of the Speech Cost/Probability bias Scale probability bias in the per-protocol analysis.*


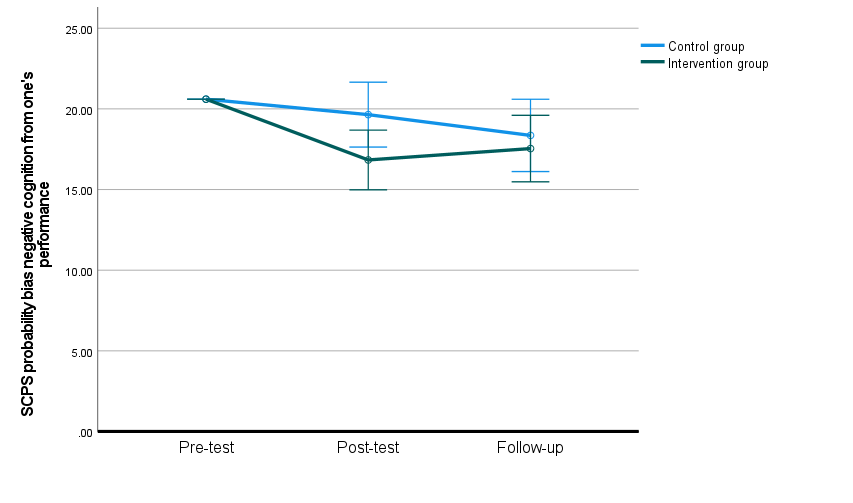


*Note.* Covariates appearing in the model are evaluated at Pre-test = 20.60; Error bars = 95% confidence interval; SCPS = Speech Cost/Probability bias Scale.

**Figure S9**

*Changes in the negative cognition generated when paying attention to others score of the Speech Cost/Probability bias Scale probability bias in the per-protocol analysis.*


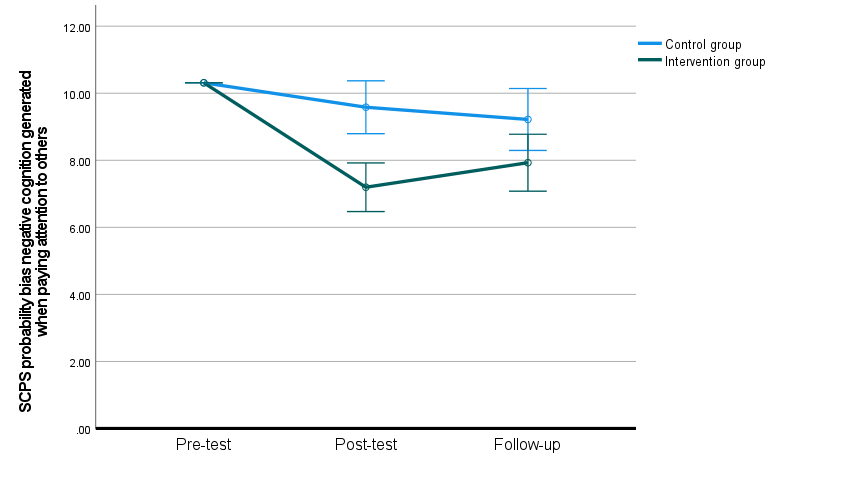


*Note.* Covariates appearing in the model are evaluated at Pre-test = 10.31; Error bars = 95% confidence interval; SCPS = Speech Cost/Probability bias Scale.

**Figure S10**

*Changes in the total score of the Self-Focused Attention scale in the per-protocol analysis.*


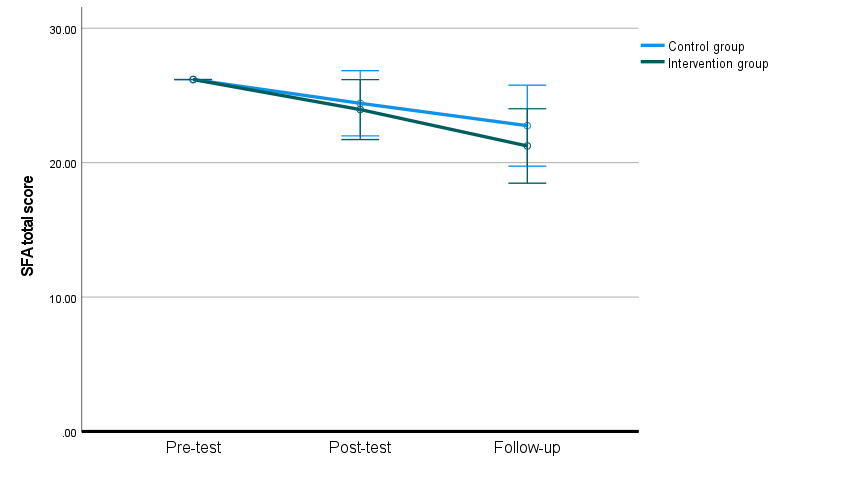


*Note.* Covariates appearing in the model are evaluated at Pre-test = 26.19; Error bars = 95% confidence interval; SFA = Self-Focused Attention scale.

**Figure S11**

*Changes in the arousal score of the Self-Focused Attention scale in the per-protocol analysis.*


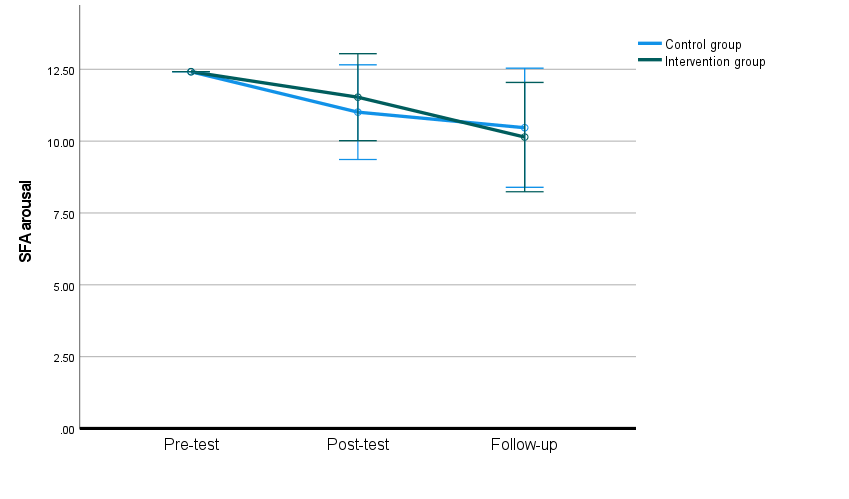


*Note.* Covariates appearing in the model are evaluated at Pre-test = 12.42; Error bars = 95% confidence interval; SFA = Self-Focused Attention scale.

**Figure S12**

*Changes in the behavior score of the Self-Focused Attention scale in the per-protocol analysis.*


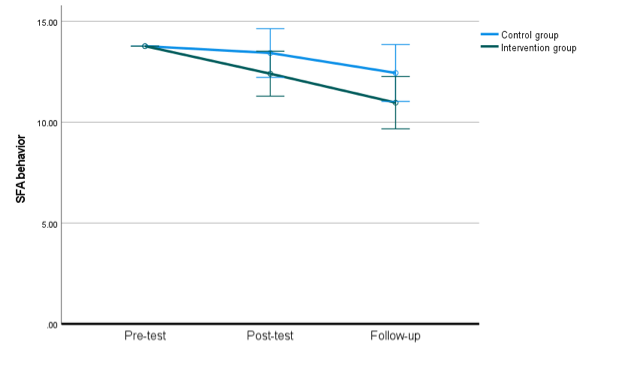


*Note.* Covariates appearing in the model are evaluated at Pre-test = 13.77; Error bars = 95% confidence interval; SFA = Self-Focused Attention scale.

**Figure S13**

*Changes in the total score of the Short Fear of Negative Evaluation scale in the per-protocol analysis.*


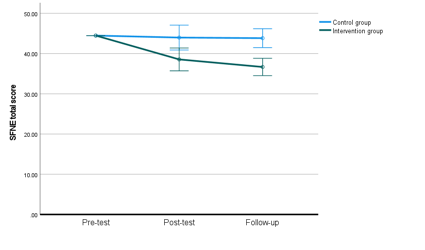


*Note.* Covariates appearing in the model are evaluated at Pre-test = 44.48; Error bars = 95% confidence interval; SFNE = Short Fear of Negative Evaluation scale.

**Figure S14**

*Changes in the forward-item score of the* *Short Fear of Negative Evaluation scale in the per-protocol analysis.*


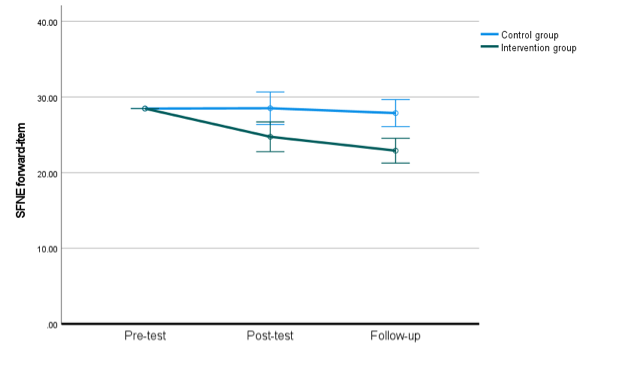


*Note.* Covariates appearing in the model are evaluated at Pre-test = 28.48; Error bars = 95% confidence interval; SFNE = Short Fear of Negative Evaluation scale.

**Figure S15**

*Changes in the reversed-item score of the* *Short Fear of Negative Evaluation scale in the per-protocol analysis.*


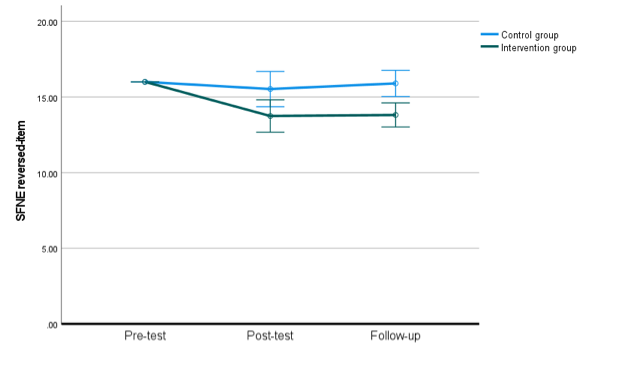


*Note.* Covariates appearing in the model are evaluated at Pre-test = 16.00; Error bars = 95% confidence interval; SFNE = Short Fear of Negative Evaluation scale.

**Figure S16**

*Changes in the total score of the Five Facet Mindfulness Questionnaire in the per-protocol analysis.*


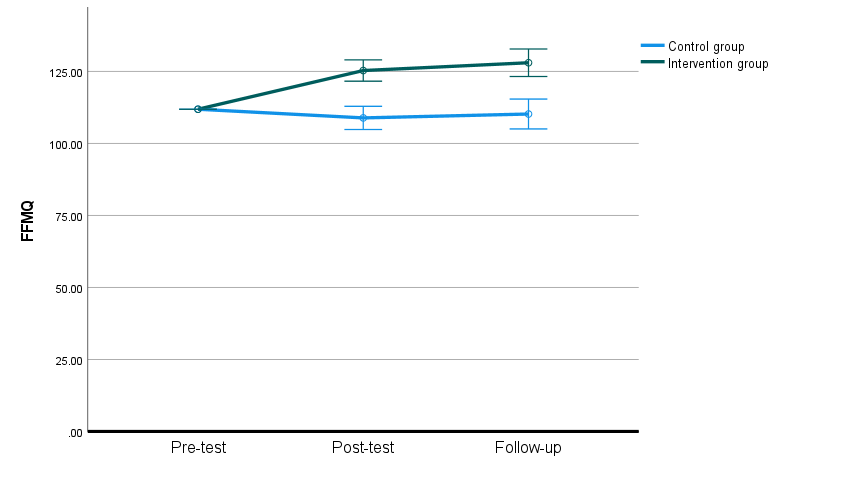


*Note.* Covariates appearing in the model are evaluated at Pre-test = 111.90; Error bars = 95% confidence interval; FFMQ = Five Facet Mindfulness Questionnaire.

**Figure S17**

*Changes in the total score of the Self-rating Depression Scale in the per-protocol analysis.*


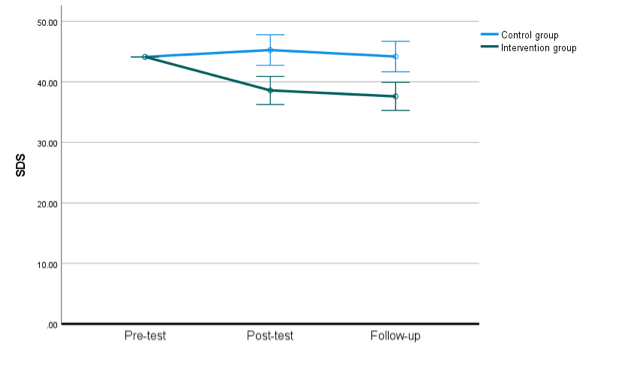


*Note.* Covariates appearing in the model are evaluated at Pre-test = 44.13; Error bars = 95% confidence interval; SDS = Self-rating Depression Scale.

**Figure S18**

*Changes in the total score of the Subjective Happiness Scale in the per-protocol analysis.*


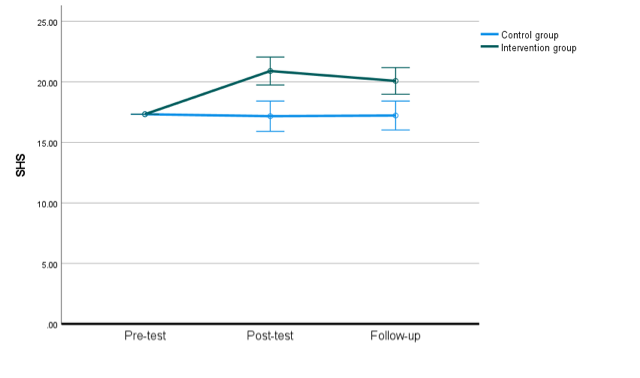


*Note.* Covariates appearing in the model are evaluated at Pre-test = 17.33; Error bars = 95% confidence interval; SHS = Subjective Happiness Scale.
